# Identification and Profiling of microRNAs Expressed in Elongating Cotton Fibers Using Small RNA Deep Sequencing

**DOI:** 10.3389/fpls.2016.01722

**Published:** 2016-11-17

**Authors:** Yanmei Wang, Yan Ding, Jin-Yuan Liu

**Affiliations:** Laboratory of Plant Molecular Biology, Center for Plant Biology, School of Life Sciences, Tsinghua UniversityBeijing, China

**Keywords:** cotton, microRNAs, fiber elongation, high-throughput sequencing, expression profile, functional network, target gene, cleavage site

## Abstract

Plant microRNAs (miRNAs) have been shown to play essential roles in the regulation of gene expression. In this study, small RNA deep sequencing was applied to explore novel miRNAs expressed in elongating cotton fibers. A total of 46 novel and 96 known miRNAs, primarily derived from the corresponding specific loci in genome of *Gossypium arboreum*, were identified. 64 miRNAs were shown to be differentially expressed during the fiber elongation process; 16 were predicted to be novel miRNAs while the remaining 48 belong to known miRNA families. Furthermore, RLM-5′ RACE (RNA ligase-mediated rapid amplification of 5′-cDNA ends) experiments identified the targets of eight important miRNAs, and the expression levels of these target genes were confirmed to be negatively correlated with the expression patterns of their corresponding miRNAs. We propose a potential functional network mediated through these eight miRNAs to illustrate their important functions in fiber elongation. Our study provides novel insights into the dynamic profiles of these miRNAs and a basis for investigating the regulatory mechanisms involved in the elongation of cotton fibers.

## Introduction

Upland cotton (*Gossypium hirsutum*) is a widely cultivated economic crop that provides natural textile fibers and material for edible oil. A cotton fiber is a single-cell trichome derived from the epidermal cells of the ovule, and its development occurs in four overlapping stages: initiation, elongation, secondary wall thickening, and maturation (Qin and Zhu, 2011; Du et al., 2013; Tang et al., 2016). Following initiation on the day of anthesis, single-celled seed trichomes subsequently undergo endoduplication and rapid elongation (Kim and Triplett, 2001; Yang et al., 2008). Thus, cotton fiber is a powerful model for studying cell differentiation and elongation.

Small RNAs (sRNAs), ranging from 18 to 24 nt in length, are key post-transcriptional regulators of gene expression in many eukaryotic organisms. A distinct class of sRNAs, known as microRNAs (miRNAs), negatively regulates gene expression by either mRNA degradation or translational inhibition. Advances in the understanding of miRNA-mediated gene regulation revealed that miRNAs play important roles in plant developmental switching and plant responses to environmental abiotic and biotic stresses as well as signal transduction (Jones-Rhoades et al., 2006; Li and Zhang, 2016). To date, several studies have investigated many miRNAs and their regulatory functions in the developmental processes of cotton fibers (Wang et al., 2012, 2015; Xue et al., 2013; Liu et al., 2014; Yu et al., 2014; Xie et al., 2015). Both computational prediction and experimental examinations have been widely employed to identify miRNAs in plants. From the very beginning, the identification of cotton miRNAs depended on a homolog-based comparative genomics approach, in which new miRNAs are identified by the comparison of currently known mature miRNAs or pre-miRNAs sequences with all cotton genomic sequences and/or ESTs (Zhang et al., 2005; Qiu et al., 2007). In 2008, two miRNAs were cloned from the cotton ovule (0–10 day post-anthesis; DPA) through sequencing (Abdurakhmonov et al., 2008). Recently, high-throughput sequencing technology has emerged as a powerful tool for identifying miRNAs and investigating their expression profiles on a genome-wide level. In 2009, four novel and 27 conserved miRNA families were identified from *G. hirsutum* L. cv. Texas Marker-1 in ovules (–3, 0, and 3 DPA) and 7 DPA fibers (Pang et al., 2009). In our previous studies, a comparative small RNAomic analysis combined with northern blotting and RACE–PCR (Rapid amplification of cDNA ends-polymerase chain reaction) was performed to reveal seven fiber initiation-related miRNAs in developing cotton ovules (Wang et al., 2012). Subsequently, eight fiber elongation-related miRNAs were identified in the genome of *G. raimondii* Ulbr (Xue et al., 2013). Recently, 21 novel miRNA candidates were found to be expressed in secondary wall thickening fiber cells (Yu et al., 2014) and 23 novel miRNAs were identified in developing cotton seeds (Wang et al., 2015). To date, a total of 713 and 427 miRNAs have been identified in the model plant species *Oryza sativa* and *Arabidopsis thaliana*, respectively. However, only 378 miRNAs from *Gossypium spp*. have been registered in miRBase (release 21). Therefore, the potential to discover novel miRNAs related to fiber development is immense.

With the goal to identify novel miRNAs in elongating fiber cells, we generated and sequenced four small RNA libraries from 5, 10, 15, and 20 DPA elongating cotton fibers. A comprehensive view of the variations in the expression patterns of miRNAs in the four samples was obtained based on high-throughput sequencing and published *G. arboreum* genome (Li et al., 2014). A total of 96 known and 46 novel miRNAs with 1127 genes as targets, were identified, of which 64 miRNAs were differentially expressed. The verification of eight elongation-related miRNAs and their putative targets by RLM-5′ RACE (RNA ligase-mediated rapid amplification of 5′-cDNA ends) experiments confirmed the sequencing results. Subsequently, qRT-RCR was used to verify the expression pattern of these miRNAs and their putative targets to illustrate the roles of miRNAs during cotton fiber elongation.

## Materials and Methods

### Plant Material Preparation and Total RNA Isolation

Seeds of upland cotton (*G. hirsutum* cv. CRI35) were kindly provided by Cotton Research Institute of Chinese Academy of Agricultural Sciences. The seeds were germinated and maintained in pots under greenhouse conditions at 28°C/25°C (day/night) with 60% relative humidity and a photoperiod of 16/8 h (day/night) for 20 days. Then, the well-grown plantlets were transplanted to an open field at Tsinghua University in Beijing to continue growing. Flowers were tagged on the day of anthesis. Normal bolls were harvested at 5, 10, 15, and 20 DPA and temporarily stored on ice. The young seeds with fibers were stripped of hulls, frozen in liquid nitrogen and stored at –80°C. Total RNA was extracted from the frozen 5, 10, 15, and 20 DPA fibers using the PureLink^TM^ Plant RNA Reagent kit (Invitrogen, USA) according to the manufacturer’s instructions. The quality of RNA was tested using an Agilent 2100 Bioanalyzer (Agilent Technologies, USA).

### Small RNA Library Construction and Sequencing

Small RNA library construction was performed as described previously (Wang et al., 2012). Briefly, 15–30 nt small RNAs were isolated on 15% PAGE (7 M urea), and ligated to the 5′ and 3′ RNA adaptors. Then, RT-PCR using primers with partial complementarity to the adaptors was performed. Four DNA pools from different samples were amplified from the first-strand cDNA and then sequenced using Hiseq2000 (Illumina, USA) at the Beijing Genomics Institute (BGI), Shenzhen, China.

### Prediction of Known and Novel miRNAs

The raw reads from the small RNA libraries were first filtered to remove low-quality reads and the adaptor sequences were trimmed to obtain clean reads. After removing sequences belonging to rRNA, tRNA, snRNA, and snoRNA in the Rfam database^1^, high-quality small RNA reads ranging from 18 to 28 nt were aligned against the genome of *G. arboreum*^2^ and reads mapping to the cotton genome sequence assemblies were retained for further analysis. Subsequently, the unique reads that could be mapped onto miRNA precursors and mature miRNAs in miRBase (release 21: June 2014^3^) were considered known miRNAs with the maximum of two mismatches. The remaining sequences were analyzed for predictions to identify novel miRNA candidates using the mireap_0.2 program^4^. Briefly, the cotton genome was used as a reference to explore the potential precursors for novel miRNA candidates in *G. arboreum*, and the obtained precursor sequences were examined for the potential to form secondary structures. The secondary structures were further checked for free energy, dominance of the novel miRNA reads relative to other precursor-mapped small RNA reads in abundance, the number of mismatches between the miRNA and the other arm of the hairpin, and no more than two asymmetric bulges in the stem region, to meet the gold criteria defined previously for the annotation of plant miRNAs (Meyers et al., 2008). We calculated the minimal folding free energy index (MFEI) of all candidates to confirm whether they were true miRNAs. Based on previous studies, the value of MFEI for most potential precursors was greater than 0.85, which is remarkably higher than that of other non-coding small RNAs. The MFEI was calculated as (100 × MFE)/(length × G/C content) (Zhang et al., 2006). Secondary structures of the obtained miRNA sequences were predicted using MFOLD^5^.

### Analysis of Differentially Expressed miRNAs

Normalized reads (reads per 10 million, RPTM; defined as actual miRNA count/total count of clean reads ^∗^1,000,000) were used to evaluate the relative expression levels of each miRNA. Differentially expressed miRNAs were calculated according to our previous publications (Wang et al., 2012; Xue et al., 2013). Forty-eight known miRNAs and 16 novel miRNAs, with normalized expression levels more than 100 RTPM in at least one library, were selected to perform cluster analysis. A hierarchical clustering algorithm implemented in the Genesis software^6^ was applied to determine the miRNAs related to fiber elongation in cotton fibers.

### Target Gene Prediction

The targets of miRNAs were predicted using the web server psRNATarget^7^ with the default parameters (Dai and Zhao, 2011). The most abundant sequence from each miRNA family served as the query, and the *Gossypium* (cotton) DFCI Gene index (CGI) Release 11^8^ as the sequence database for the target search.

### Verification of the miRNA Cleavage Sites Using RLM-5′ RACE

To identify the cleavage sites of target transcripts, the First Choice RLM-RACE kit (Ambion, USA) was used to perform RLM-5′ RACE. Total RNA was extracted using the PureLink^TM^ Plant RNA Reagent (Invitrogen) from fibers (5, 10, 15, and 20 DPA), and poly(A)^+^ mRNA were purified using a mRNA Purification Kit (Invitrogen). The purified mRNA was reverse transcribed to cDNA using the TaKaRa RNA PCR Kit. The resulting samples were ligated to a 5′ RACE adapter. Gene-specific reverse primers and gene-specific reverse nested primers were designed for the predicted targets and used in combination with the 5′ RACE adapter primers to amplify the cleaved transcripts. The 5′ RACE products were purified, cloned into pEASY-T1 vector (TransGen, Beijing) and sequenced. All gene-specific primers used in the experiments are listed in Supplementary Table S8.

### Expression Analysis of Target Genes Using Quantitative Real-Time RT-RCR (qRT-RCR)

Total RNAs were extracted using the PureLink^TM^ Plant RNA Reagent (Invitrogen) from fibers (5, 10, 15, and 20 DPA). cDNAs were synthesized from 2 μg of total RNA using the TaKaRa RNA PCR Kit. Quantitative RT-PCR was performed on an iCycler iQ5 Multicolor real-time PCR detection system (Bio-Rad) using the Power SYBR Green PCR MasterMix (Applied Biosystems). Each PCR reaction (10 μL) contained 5 μL of real-time PCR Mix, 0.1 μL of each primer and the appropriately diluted cDNA. The thermal cycling conditions were 95°C for 30 s followed by 40 cycles of 95°C for 10 s, 55°C for 30 s, and 72°C for 15 s. The *GhUBQ10* transcript was used as an internal reference for all the qRT-PCR analyses (Walford et al., 2011). Each sample was analyzed using three biological replicates and three technical replicates for each biological replicate. The relative gene expression levels were calculated according to the 2^-ΔΔCT^ method of the system. The primers used are described in Supplementary Table S8.

## Results and Discussion

### Deep Sequencing and Data Analysis

To identify novel miRNAs in elongating cotton fibers, we constructed four small RNA libraries from allotetraploid cotton fibers at 5, 10, 15, and 20 DPA. The small RNA libraries were sequenced on an Illumina HiSeq 2000 analyzer. A total of 76.2 million raw reads were generated, and the total numbers of clean reads, ranging from 18 to 44 nucleotides in length, obtained from each of the four libraries after precluding low quality reads, adaptor contamination and RNAs smaller than 18 nucleotides were as follows: 17979849 (F5), 18747051 (F10), 19597538 (F15), and 17845500 (F20) (Supplementary Table S1). These high-quality sRNAs were used for further analyses. Our analyses showed that 2338626, 2350998, 2146087, and 1581403 of the clean reads in the F5, F10, F15, and F20 libraries, respectively, were aligned to the *G. arboreum* genome (**Table 1**). In addition, sequences corresponding to rRNAs, tRNAs, snRNAs, and snoRNAs were also identified (**Table 1**).

**Table 1:** Distribution of small RNA sequences in the elongating cotton fiber small RNA libraries.

RNA class	5 DPA		10 DPA		15 DPA		20 DPA	
Clean reads	7334102	100%	7110884	100%	6490626	100%	4877186	100%
Match to genome^a^	2338626	31.89%	2350998	33.06%	2146087	33.06%	1581403	32.42%
rRNA	52257	0.71%	88323	1.24%	105694	1.63%	162270	3.33%
tRNA	2913	0.00%	5568	0.00%	6599	0.00%	11615	0.01%
snRNA	83	0.00%	156	0.00%	188	0.00%	268	0.00%
snoRNA	14	0.04%	18	0.08%	28	0.10%	170	0.24%
Repeats	2327	0.03%	2588	0.04%	2568	0.04%	2287	0.05%
No annotation	4937882	67.33%	4663233	65.58%	4229462	65.16%	3119173	63.95%

*^*a*^Mapped to the *G. arboreum* whole genome sequence (http://cgp.genomics.org.cn/page/species/index.jsp).*

**FIGURE 1:**
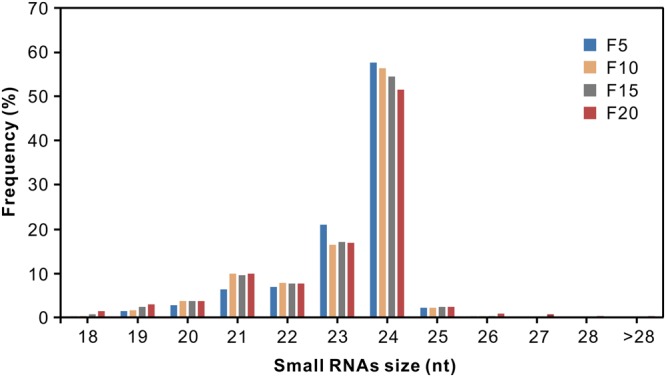
**Sequence length distribution of small RNAs from the elongating cotton fiber libraries isolated at 5, 10, 15, and 20 DPA**.

Of the millions of high-quality sRNAs obtained, 95.27% (F5), 94.98% (F10), 92.96% (F15), and 90.24% (F20) were 20–24 nt in length with 24, 23, and 21 nt sequences representing the predominant size classes (**Figure 1**; Supplementary Table S1). It has been reported that most sRNAs functioning as small interfering RNAs (siRNAs) are 24 nt long (Rajagopalan et al., 2006); the appearance of such large numbers of 24 nt sRNAs in the four small RNA libraries indicated that siRNAs accounted for a large proportion in the sRNA datasets. These results are in agreement with previous reports on cotton (Wang et al., 2015) and other plants such as rice (Li et al., 2011), maize (Kang et al., 2012), and *Medicago truncatula* (Szittya et al., 2008).

### Identification of Known and Novel miRNAs

Currently, 378 unique mature sequences, belonging to 217 families registered in miRBase (release 21: June 2014^9^) for cotton. To identify conserved miRNAs in the four libraries, the candidate miRNAs sequences were compared with those of published miRNAs using BLASTN search in miRBase. In total, 96 known miRNAs, belonging to 45 conserved miRNA families, were found to be expressed during cotton fiber elongation (Supplementary Table S2). Another previous study (Pang et al., 2009) also revealed 27 conserved and 4 novel miRNA families in four sequencing libraries from seedling leaves and ovules at -3, 0, and 3 DPA. Then, miRNA microarrays have been employed to measure expression of 21 conserved miRNAs present in their sequencing libraries and further found an additional nine miRNAs expressed in 7 DPA fibers. In our study, the 45 miRNA families included not only nearly all the miRNA families previously identified by Pang et al. (2009) but also another 18 known miRNA families in miRBase.

Another notable feature in this study is that the number of miRNAs varied among identified families (Supplementary Table S2). The miR156 and miR166 family were the two largest identified families, with seven members distinguished by internal nucleotide diversity or different genomic locations of pre-miRNAs. The miRNA families of miR169, miR482, miR157, miR160, miR164, miR167, miR390, and miR399 were represented by 6, 5, 4, 4, 4, 4, 4, and 4 members, respectively. Among the remaining 35 miRNA families, 11 were represented by two to three members and 24 by only one member. It was also observed that many sequence-variants were identified for almost every member of the known miRNAs.

One of the most important advantages of high-throughput sequencing technology is that it can facilitate the detection of novel miRNAs with extremely low expression level. Following the three criteria suggested by Meyers et al. (2008), a total of 46 novel miRNAs represented by 45 unique miRNA sequences were predicted (Supplementary Table S3). These novel miRNA candidates were named temporarily using the GhmiRna-number format, before being submitted for an official designation. The lengths of the newly identified miRNAs ranged from 19 to 24 bp; 21 nt miRNAs comprised the largest category followed by 24 nt miRNAs, and other species were much less frequent (**Table 2**), which is consistent with the observation that miRNAs are typically 21 or 24 nt in plants. Additionally, first nucleotide bias analysis of novel miRNAs showed that adenosine (A, 39.1%) and uracil (U, 32.6%) were the two most prominent nucleotides at the 5′ terminus (**Table 2**), which is in agreement with previous findings that AGO4 displays a preference for miRNAs with 5′ terminal A, while AGO1 usually recruits miRNAs with 5′ terminal U (Mi et al., 2008).

**Table 2:** The 5′ terminal nucleotides and lengths of the 46 novel cotton miRNAs.

5′ terminal	miRNA length (nt)						
	19	20	21	22	23	24	Total
A	0	0	3	3	2	10	18
U	0	0	13	2	0	0	15
C	0	0	3	0	0	3	6
G	1	0	1	1	0	4	7
Total	1	0	20	6	2	17	46

The precursor of each novel miRNA was able to form an appropriate secondary hairpin structure with the minimal folding free energy (MFE) ranging from -24.1 to -66.5 kcal/mol (Supplementary Figure S1; Supplementary Table S3). Furthermore, the MFEI, an important parameter to distinguish miRNAs from other non-coding RNAs, defines that a candidate RNA sequence is more likely to be a miRNA when the MFEI is greater than 0.85. Hence, we calculated the MFEI for each novel miRNA sequence (Supplementary Table S3), and found that 30 of the 46 novel miRNA sequences exhibited a MFEI greater than 0.85, while the MFEIs of 16 miRNAs were lower than 0.85. These results are in agreement with previous studies (Bonnet et al., 2004; Zhang et al., 2006).

### Expression of Known and Novel miRNAs during Cotton Fiber Elongation

To measure miRNA read abundance, the abundances of known miRNAs were normalized to reads per 10 million (RPTM). In the four cotton fiber sRNA libraries, there were 94 known miRNAs in common, while miR398 was only found in the F15 and F20 libraries; another miRNA, GhmiR5141, was undetected in the F20 library (Supplementary Figure S2A; Supplementary Table S4). Further analysis found that 38 of the known miRNAs exhibited average abundances of more than 100 RPTM in the F5 library, 46 in the F10 library, 42 in the F15 library, and 45 in the F20 library (Supplementary Figure S2C). However, all the 46 predicted novel miRNAs were expressed in all four libraries, while 10 were highly expressed in the F5 library, 11 in the F10 library, 14 in the F15 library, and 13 in the F20 library (Supplementary Figures S2B,D; Supplementary Table S5).

**FIGURE 2:**
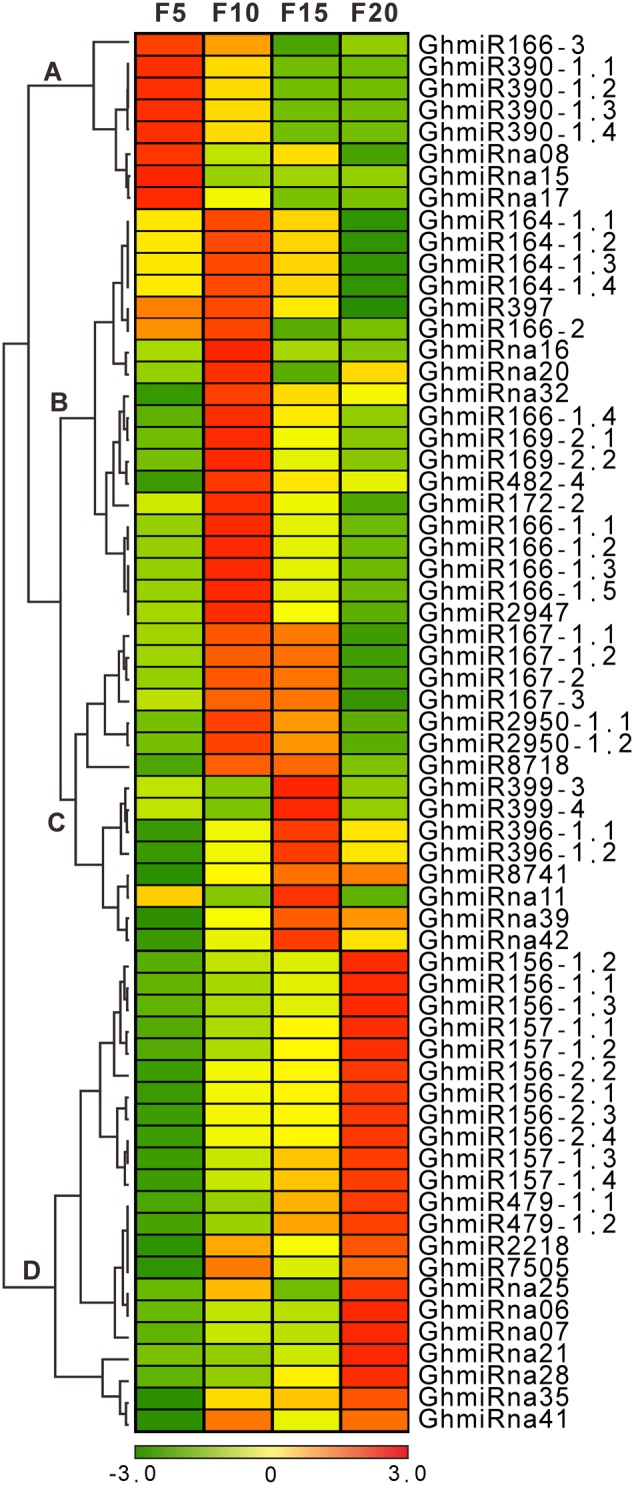
**A complete linkage hierarchical cluster analysis of 16 novel and 48 known differentially expressed miRNAs in the cotton fibers was performed by comparing the RPTM of the miRNAs in every library to the average of the four cotton fiber sRNA libraries (5, 10, 15, and 20 DPA)**. The color indicates the log_2_-fold change from high (red) to low (green), as indicated in the color scale. On the right are names of the miRNAs, on the left are the classes to which they belong.

The 48 known and 16 novel strongly expressed miRNAs, with more than 100 RPTM in at least one library, were used to perform cluster analyses. Based on the hierarchical clustering method, the expression patterns of miRNAs were clustered into four classes in both known and novel miRNAs (**Figure 2**). In class A, two known miRNA families (GhmiR166 and GhmiR390) and three novel miRNAs (GhmiRna08, GhmiRna15, and GhmiRna17) were progressively down-regulated during cotton fiber elongation, indicating that these miRNAs may play important negative regulatory roles in fiber elongation after 5 DPA. Consistently with previous findings of a general repression of miRNAs in fibers (Pang et al., 2009; Wang et al., 2012), we found that GhmiR166 and GhmiR390 were down-regulated along with fiber initiation, which correlates with the up-regulation of a dozen miRNA targets that promote fiber initiation. The abundance of two known miRNA families (GhmiR164 and GhmiR397) and three novel miRNAs (GhmiRna16, GhmiRna20, and GhmiRna32) in class B was very high at 10 DPA. Class C consisted of six known miRNAs (GhmiR167, GhmiR2950, GhmiR8718, GhmiR399, GhmiR396, and GhmiR8741) and three novel miRNAs (GhmiRna11, GhmiRna39, and GhmiRna42), whose accumulation peaked at 10 or 15 DPA during the rapid fiber elongation period (Ruan et al., 2001; Zhang and Liu, 2016), implying that these miRNAs may promote rapid fiber elongation. Combined with previously studies (Pang et al., 2009; Wang et al., 2012), GhmiR164 and GhmiR167 may function not only in fiber-bearing ovules (3 DPA) but also in rapid elongating cotton fibers. In class D, the abundance of the five known miRNA families (GhmiR156, GhmiR157, GhmiR479, GhmiR2218, and GhmiR7505) and seven novel miRNAs continuously increased during cotton fiber elongation, particularly at 20 DPA, the time at which fiber elongation is terminated.

### Targets of Known and Novel miRNAs

Previous studies have indicated that miRNAs inhibit gene expression by binding to protein-coding mRNAs. To elucidate the functions of miRNAs in cotton fiber elongation, we predicted their potential regulatory targets (Supplementary Tables S6 and S7) using the psRNATarget program. As shown in Supplementary Table S6, 729 non-redundant EST (expressed sequence tag) sequences, with an average of 7.8 targets/miRNA was predicted for 94 known miRNAs. On the other hand, a total of 451 EST sequences that matched the 43 novel miRNAs are listed in Supplementary Table S7. No target genes were found for the remaining two known miRNAs (GhmiR166-3 and GhmiR8682) and three novel miRNAs (GhmiRna02, GhmiRna18, and GhmiRna28), suggesting that the miRNAs with no predicted targets may suppress gene expression by different mechanisms including the inhibition of translation. Most miRNA families have multiple distinct targets, indicating that these miRNAs may play numerous roles in cotton fiber development. On the other hand, a single gene may also be targeted by several miRNAs, which map to the same cDNA at different sites, and cleave the mRNA into different-sized fragments. In addition, miRNAs in one family were found to target the same mRNAs.

### Cleavage Sites and Expression Correlation between miRNAs and Their Targets

**FIGURE 3:**
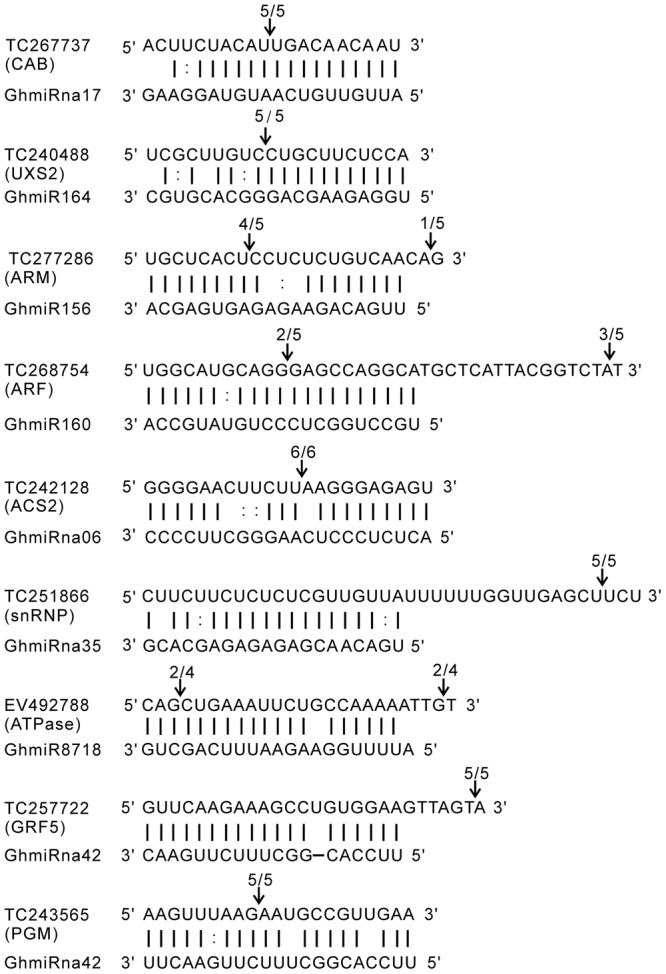
**Mapping of target mRNA cleavage sites by RLM-5′ RACE.** The arrows indicate the cleavage sites and the numbers show the fraction of cloned PCR products terminating at different positions. TC267737: *CAB* (chlorophyll a/b binding protein CP29); TC240488: *UXS2* (UDP-glucuronic acid decarboxylase 2); TC277286: *ARM* (armadillo repeat superfamily protein); TC268754: *ARF* (auxin response factor); TC242128: *ACS2* (1-aminocyclopropane-1-carboxylic acid synthase); TC251866: *snRNP* (small nuclear ribonucleoprotein family protein); EV492788: *ATPase* (actin-like ATPase superfamily protein); TC257722: *GRF5* (growth-regulating factor 5); TC243565: *PGM* (phosphoglycerate mutase).

The RLM-5′ RACE experiment was carried out to verify the cleavage sites of the predicted miRNA target genes. The targets genes of the four known (GhmiR156, GhmiR160, GhmiR164, and GhmiR8718) and four novel miRNAs (GhmiRna06, GhmiRna17, GhmiRna35, and GhmiRna42) were used for the following analysis. The nine target genes were TC267737 (chlorophyll a/b binding protein CP29, *CAB*), TC240488 (UDP-glucuronic acid decarboxylase 2, *UXS2*), TC277286 (armadillo repeat superfamily protein, *ARM*), TC268754 (auxin response factor, *ARF*), TC242128 (1-aminocyclopropane-1-carboxylic acid synthase, *ACS2*), TC251866 (small nuclear ribonucleoprotein family protein, *snRNP*), EV492788 (actin-like ATPase superfamily protein, *ATPase*), TC257722 (growth-regulating factor 5, *GRF5*), TC243565 (phosphoglycerate mutase, *PGM*). All nine predicted target genes had specific cleavage sites that were located in the complementary sequences between miRNAs and their targets (**Figure 3**). *CAB, UXS2, ARF*, and *ACS2* were precisely mapped from the 9–11th position of complementary sequences at the 5′ end of the miRNA, showing evidence of cleavage by GhmiRna17, GhmiR164, GhmiR160, and GhmiRna06, respectively. On the other hand, some miRNAs had multiple cleavage sites with different relative activities (**Figure 3**). For example, the target of GhmiR156, TC277286, predicted to be an armadillo repeat superfamily protein, has two cleavage sites with different relative activities in the complementary region, and the site with the highest activity is located at position 12th at the 5′ end of the miRNA.

**FIGURE 4:**
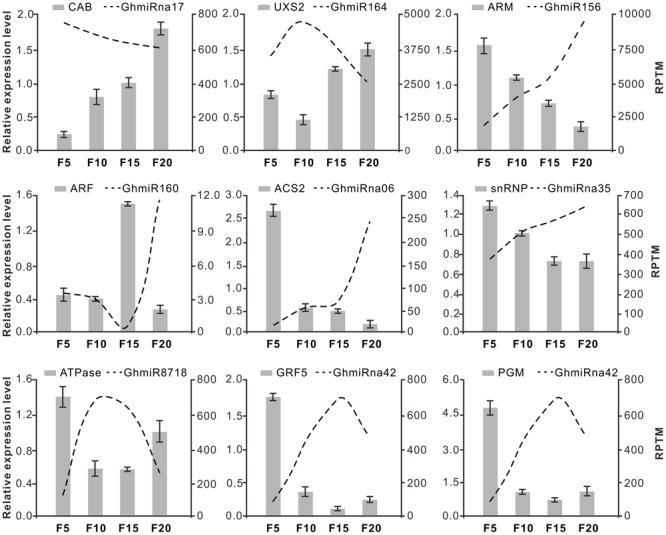
**Expression pattern correlation between miRNAs and their target genes.** The dotted lines and bars indicate miRNAs and accordingly the target abundance from the qRT-PCR results, respectively, in the four fiber libraries (5, 10, 15, and 20 DPA). The *y*-axis on the left and right were used to measure the expression levels of target and miRNA, respectively. The data represent the mean values ± SD of three replicates.

To assess the influence of the miRNAs on their targets, quantitative RT-PCR (qRT-PCR) analysis was used to quantify the transcription levels of the nine target genes during cotton fiber elongation. As shown in **Figure 4**, for the targets of Class A miRNAs, the transcript levels of *CAB* were negatively correlated with the reduction in GhmiRna17 levels. In contrast to GhmiRna17 expression, which decreased from 5 to 20 DPA, the expression of *CAB* increased from 5 to 20 DPA. It was reported that CAB proteins constitute the major light harvesting complex, which facilitate at the initial capture of light energy (Stayton et al., 1986; Anderson et al., 1993). This result indicated that GhmiRna17 may be associated with energy metabolism during fiber development. Among the targets of Class B miRNAs, the expression levels of which increased from 5 DPA and reached peak levels at 10 DPA, the expression of *UXS2* was reduced from 5 to 10 DPA and subsequently up-regulated at 20 DPA (**Figure 4**). Interestingly, UXS2 has been found to be closely correlated with UDP-xylose synthesis, a process that is critical to the development of primary walls in fiber cells (Pan et al., 2010). Thus, GhmiR164 may serve as a key regulator of fiber elongation. The expression of GhmiR156 increased from 5 to 20 DPA, which might contribute to suppressing the expression of its target gene *ARM* (**Figure 4**). The same phenomenon was observed with between GhmiRna35 and its target *snRNP*; while snRNPs are implicated in pre-mRNA splicing and mature mRNA formation (Zhang et al., 2007). Previous investigations showed that the *ARM* genes function in several cellular processes including signal transduction, cytoskeletal regulation, nuclear import, transcriptional regulation, and ubiquitination (Phillips et al., 2012; Sharma and Pandey, 2015). Additionally, low expression of GhmiR160 was found to lead to the accumulation of ARFs at 15 DPA (**Figure 4**), which act as transcriptional activators and repressors that bind to the auxin response elements to regulate the expression of other genes (Tiwari et al., 2003). It has been reported that auxin promotes the development of fiber cells *in vitro* cultured ovules, and displays a positive correlation between final fiber length and *in vivo*-quantified IAA levels (Gokani and Thaker, 2002). Hence, GhmiR160, via its mediation of the cleavage of *ARF* mRNA, might be critical for rapid fiber elongation. The reads of GhmiRna06 increased gradually from 5 to 20 DPA and were negatively correlated with the level of ACS2 expression (**Figure 4**). It is also worth mentioning that ACS is the rate-limiting enzyme in ethylene biosynthesis during cotton fiber elongation (Wang et al., 2011). The expression levels of Class C miRNAs (GhmiR8718 and GhmiRna42) increased from 5 DPA and peaked at 10 or 15 DPA, while those of their target genes were reduced from 5 to 10 DPA or 15 DPA and slightly increased at 20 DPA. GhmiR8718, whose target mRNA encodes an ATPase, might be involved in cell expansion during elongation (Joshi et al., 1988). Two GhmiRna42 targets, *GRF5* and *PGM*, were also identified (**Figure 4**); GRFs are plant-specific transcription factors that have diverse growth-related functions (Omidbakhshfard et al., 2015), and PGM is a ubiquitous glycolytic enzyme that plays an important role in glycogen metabolism in eukaryotic cells (Zhao and Assmann, 2011; Jablonsky et al., 2013).

**FIGURE 5:**
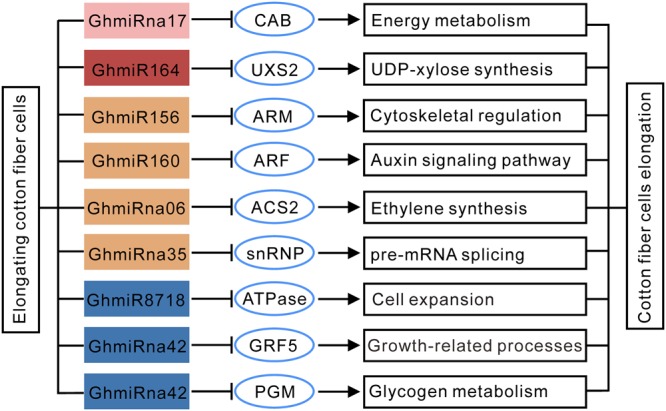
**A possible functional network of fiber elongation-related miRNAs in cotton fiber.** The arrows indicate positive regulation, and the nail shapes represent negative regulation.

To confirm the results of qRT-PCR experiments, we analyzed the RNA-seq data from *G. hirsutum* transcriptome analyses downloaded from the NCBI Sequence Read Archive (SRA) under the accession number PRJNA248163. Consistent expression patterns of these nine miRNA target genes during cotton fiber elongation were observed between both qRT-PCR and RNA-seq-based differential expression analysis (Supplementary Figure S3). Therefore, our results demonstrate that miRNA-mediated regulation occurs in response to fiber elongation. A putative regulatory network connecting fiber elongation-related miRNAs to their targets may play a crucial role in cotton fiber elongation (**Figure 5**).

## Conclusion

In summary, a total of 96 known miRNAs and 46 novel miRNAs were identified through the high-throughput sequencing of sRNAs isolated from 5, 10, 15, and 20 DPA cotton fibers. A complete linkage hierarchical cluster analysis revealed that 48 known miRNAs and 16 novel miRNAs were differentially expressed during cotton fiber elongation. Further, the target genes of eight miRNAs were validated through RLM-5′ RACE. The expression patterns of these target genes revealed negative correlation with the expression levels of their corresponding miRNAs. Thus, the present study revealed a regulatory network of miRNAs and may further facilitate the investigation of the functional importance of miRNA-mediated gene regulation during fiber elongation.

## Author Contributions

J-YL conceived and designed the study. YW analyzed the sequencing data. YW and YD performed RLM-5′ RACE and qRT-PCR. J-YL, YW, and YD wrote the manuscript. All the authors read and approved the final manuscript.

## Conflict of Interest Statement

The authors declare that the research was conducted in the absence of any commercial or financial relationships that could be construed as a potential conflict of interest.

## Footnotes

1 http://rfam.xfam.org

2 http://cgp.genomics.org.cn/page/species/index.jsp

3 http://microrna.sanger.ac.uk

4 http://sourceforge.net/projects/mireap

5 http://unafold.rna.albany.edu/?q=mfold

6 http://genome.tugraz.at/

7 http://plantgrn.noble.org/psRNATarget/

8 http://compbio.dfci.harvard.edu/index.html

9 http://microrna.sanger.ac.uk

## References

[B1] Abdurakhmonov I. Y. Devor E. J. Buriev Z. T. Huang L. Makamov A. Shermatov S. E. (2008). Small RNA regulation of ovule development in the cotton plant, *G. hirsutum* L. *BMC Plant Biol.* 8:93 10.1186/1471-2229-8-93 PMC2564936 18793449

[B2] Anderson D. M. Hudspeth R. L. Hobbs S. L. Grula J. W. (1993). Chlorophyll a/b-binding protein gene expression in cotton. *Plant Physiol.* 102 1047–1048. 10.1104/pp.102.3.1047 8278525 PMC158882

[B3] Bonnet E. Wuyts J. Rouze P. Van De Peer Y. (2004). Evidence that microRNA precursors, unlike other non-coding RNAs, have lower folding free energies than random sequences. *Bioinformatics* 20 2911–2917. 10.1093/bioinformatics/bth374 15217813

[B4] Dai X. Zhao P. X. (2011). psRNATarget: a plant small RNA target analysis server. *Nucleic Acids Res.* 39 W155–W159. 10.1093/nar/gkr319 21622958 PMC3125753

[B5] Du S. J. Dong C. J. Zhang B. Lai T. F. Du X. M. Liu J. Y. (2013). Comparative proteomic analysis reveals differentially expressed proteins correlated with fuzz fiber initiation in diploid cotton (*Gossypium arboreum* L.). *J. Proteomics* 82 113–129. 10.1016/j.jprot.2013.02.020 23474080

[B6] Gokani S. J. Thaker V. S. (2002). Physiological and biochemical changes associated with cotton fiber development - IX. Role of IAA and PAA. *Field Crops Res.* 77 127–136. 10.1016/S0378-4290(02)00062-X

[B7] Jablonsky J. Hagemann M. Schwarz D. Wolkenhauer O. (2013). Phosphoglycerate mutases function as reverse regulated isoenzymes in *Synechococcus elongatus* PCC 7942. *PLoS ONE* 8:e58281 10.1371/journal.pone.0058281 PMC3590821 23484009

[B8] Jones-Rhoades M. W. Bartel D. P. Bartel B. (2006). MicroRNAs and their regulatory roles in plants. *Annu. Rev. Plant Biol.* 57 19–53. 10.1146/annurev.arplant.57.032905.105218 16669754

[B9] Joshi P. A. Stewart J. M. Graham E. T. (1988). Ultrastructural-localization of AtPase activity in cotton fiber during elongation. *Protoplasma* 143 1–10. 10.1007/Bf01282953

[B10] Kang M. Zhao Q. Zhu D. Yu J. (2012). Characterization of microRNAs expression during maize seed development. *BMC Genomics* 13:360 10.1186/1471-2164-13-360 PMC3468377 22853295

[B11] Kim H. J. Triplett B. A. (2001). Cotton fiber growth in planta and in vitro. Models for plant cell elongation and cell wall biogenesis. *Plant Physiol.* 127 1361–1366. 11743074 PMC1540163

[B12] Li C. Zhang B. (2016). MicroRNAs in control of plant development. *J. Cell Physiol.* 231 303–313. 10.1002/jcp.25125 26248304

[B13] Li F. Fan G. Wang K. Sun F. Yuan Y. Song G. (2014). Genome sequence of the cultivated cotton *Gossypium arboreum*. *Nat. Genet.* 46 567–572. 10.1038/ng.2987 24836287

[B14] Li T. Li H. Zhang Y. X. Liu J. Y. (2011). Identification and analysis of seven H_2_O_2_-responsive miRNAs and 32 new miRNAs in the seedlings of rice (*Oryza sativa* L. ssp. *indica*). *Nucleic Acids Res.* 39 2821–2833. 10.1093/nar/gkq1047 21113019 PMC3074118

[B15] Liu N. Tu L. Tang W. Gao W. Lindsey K. Zhang X. (2014). Small RNA and degradome profiling reveals a role for miRNAs and their targets in the developing fibers of *Gossypium barbadense*. *Plant J.* 80 331–344. 10.1111/tpj.12636 25131375

[B16] Meyers B. C. Axtell M. J. Bartel B. Bartel D. P. Baulcombe D. Bowman J. L. (2008). Criteria for annotation of plant MicroRNAs. *Plant Cell* 20 3186–3190. 10.1105/tpc.108.064311 19074682 PMC2630443

[B17] Mi S. Cai T. Hu Y. Chen Y. Hodges E. Ni F. (2008). Sorting of small RNAs into *Arabidopsis* argonaute complexes is directed by the 5′ terminal nucleotide. *Cell* 133 116–127. 10.1016/j.cell.2008.02.034 18342361 PMC2981139

[B18] Omidbakhshfard M. A. Proost S. Fujikura U. Mueller-Roeber B. (2015). Growth-regulating factors (GRFs): a small transcription factor family with important functions in plant biology. *Mol. Plant* 8 998–1010. 10.1016/j.molp.2015.01.013 25620770

[B19] Pan Y. X. Wang X. F. Liu H. W. Zhang G. Y. Ma Z. Y. (2010). Molecular cloning of three UDP-Glucuronate decarboxylase genes that are preferentially expressed in *Gossypium* fibers from elongation to secondary cell wall synthesis. *J. Plant Biol.* 53 367–373. 10.1007/s12374-010-9124-9

[B20] Pang M. Woodward A. W. Agarwal V. Guan X. Ha M. Ramachandran V. (2009). Genome-wide analysis reveals rapid and dynamic changes in miRNA and siRNA sequence and expression during ovule and fiber development in allotetraploid cotton (*Gossypium hirsutum* L.). *Genome Biol.* 10:R122 10.1186/gb-2009-10-11-r122 PMC3091316 19889219

[B21] Phillips S. M. Dubery I. A. Van Heerden H. (2012). Molecular characterization of an elicitor-responsive Armadillo repeat gene (*GhARM*) from cotton (*Gossypium hirsutum*). *Mol. Biol. Rep.* 39 8513–8523. 10.1007/s11033-012-1706-9 22714909

[B22] Qin Y. M. Zhu Y. X. (2011). How cotton fibers elongate: a tale of linear cell-growth mode. *Curr. Opin. Plant Biol.* 14 106–111. 10.1016/j.pbi.2010.09.010 20943428

[B23] Qiu C. X. Xie F. L. Zhu Y. Y. Guo K. Huang S. Q. Nie L. (2007). Computational identification of microRNAs and their targets in *Gossypium hirsutum* expressed sequence tags. *Gene* 395 49–61. 10.1016/j.gene.2007.01.034 17408884

[B24] Rajagopalan R. Vaucheret H. Trejo J. Bartel D. P. (2006). A diverse and evolutionarily fluid set of microRNAs in *Arabidopsis thaliana*. *Genes Dev.* 20 3407–3425. 10.1101/gad.1476406 17182867 PMC1698448

[B25] Ruan Y. L. Llewellyn D. J. Furbank R. T. (2001). The control of single-celled cotton fiber elongation by developmentally reversible gating of plasmodesmata and coordinated expression of sucrose and K^+^ transporters and expansin. *Plant Cell* 13 47–60. 10.1105/tpc.13.1.47 11158528 PMC102212

[B26] Sharma M. Pandey G. K. (2015). Expansion and function of repeat domain proteins during stress and development in plants. *Front. Plant Sci.* 6:1218 10.3389/fpls.2015.01218 PMC4707873 26793205

[B27] Stayton M. M. Black M. Bedbrook J. Dunsmuir P. (1986). A novel chlorophyll a/b binding (*Cab*) protein gene from petunia which encodes the lower molecular weight *Cab* precursor protein. *Nucleic Acids Res.* 14 9781–9796. 10.1093/nar/14.24.9781 3027663 PMC341335

[B28] Szittya G. Moxon S. Santos D. M. Jing R. Fevereiro M. P. Moulton V. (2008). High-throughput sequencing of Medicago truncatula short RNAs identifies eight new miRNA families. *BMC Genomics* 9:593 10.1186/1471-2164-9-593 PMC2621214 19068109

[B29] Tang K. Dong C. Liu J. (2016). Genome-wide analysis and expression profiling of the phospholipase D gene family in *Gossypium arboreum*. *Sci. China Life Sci.* 59 130–141. 10.1007/s11427-015-4916-2 26718354

[B30] Tiwari S. B. Hagen G. Guilfoyle T. (2003). The roles of auxin response factor domains in auxin-responsive transcription. *Plant Cell* 15 533–543. 10.1105/tpc.008417 12566590 PMC141219

[B31] Walford S. A. Wu Y. Llewellyn D. J. Dennis E. S. (2011). GhMYB25-like: a key factor in early cotton fibre development. *Plant J.* 65 785–797. 10.1111/j.1365-313X.2010.04464.x 21235650

[B32] Wang H. Mei W. Qin Y. Zhu Y. (2011). 1-Aminocyclopropane-1-carboxylic acid synthase 2 is phosphorylated by calcium-dependent protein kinase 1 during cotton fiber elongation. *Acta Biochim Biophys. Sin. (Shanghai)* 43 654–661. 10.1093/abbs/gmr056 21742672

[B33] Wang Y. Ding Y. Yu D. Xue W. Liu J. (2015). High-throughput sequencing-based genome-wide identification of microRNAs expressed in developing cotton seeds. *Sci. China Life Sci.* 58 778–786. 10.1007/s11427-015-4877-5 26117827

[B34] Wang Z. M. Xue W. Dong C. J. Jin L. G. Bian S. M. Wang C. (2012). A comparative miRNAome analysis reveals seven fiber initiation-related and 36 novel miRNAs in developing cotton ovules. *Mol. Plant* 5 889–900. 10.1093/mp/ssr094 22138860

[B35] Xie F. Jones D. C. Wang Q. Sun R. Zhang B. (2015). Small RNA sequencing identifies miRNA roles in ovule and fibre development. *Plant Biotechnol. J.* 13 355–369. 10.1111/pbi.12296 25572837

[B36] Xue W. Wang Z. Du M. Liu Y. Liu J. Y. (2013). Genome-wide analysis of small RNAs reveals eight fiber elongation-related and 257 novel microRNAs in elongating cotton fiber cells. *BMC Genomics* 14:629 10.1186/1471-2164-14-629 PMC3849097 24044642

[B37] Yang Y. W. Bian S. M. Yao Y. Liu J. Y. (2008). Comparative proteomic analysis provides new insights into the fiber elongating process in cotton. *J. Proteome Res.* 7 4623–4637. 10.1021/pr800550q 18823139

[B38] Yu D. Wang Y. Xue W. Fan S. Yu S. Liu J. Y. (2014). Identification and profiling of known and novel fiber microRNAs during the secondary wall thickening stage in cotton (*Gossypium hirsutum*) via high-throughput sequencing. *J. Genet. Genomics* 41 553–556. 10.1016/j.jgg.2014.08.002 25438700

[B39] Zhang B. Liu J. Y. (2016). Cotton cytosolic pyruvate kinase GhPK6 participates in fast fiber elongation regulation in a ROS-mediated manner. *Planta* 244 915–926. 10.1007/s00425-016-2557-8 27316434

[B40] Zhang B. Wang Q. Wang K. Pan X. Liu F. Guo T. (2007). Identification of cotton microRNAs and their targets. *Gene* 397 26–37. 10.1016/j.gene.2007.03.020 17574351

[B41] Zhang B. H. Pan X. P. Cox S. B. Cobb G. P. Anderson T. A. (2006). Evidence that miRNAs are different from other RNAs. *Cell Mol. Life Sci.* 63 246–254. 10.1007/s00018-005-5467-7 16395542 PMC11136112

[B42] Zhang B. H. Pan X. P. Wang Q. L. Cobb G. P. Anderson T. A. (2005). Identification and characterization of new plant microRNAs using EST analysis. *Cell Res.* 15 336–360. 10.1038/sj.cr.7290302 15916721

[B43] Zhao Z. Assmann S. M. (2011). The glycolytic enzyme, phosphoglycerate mutase, has critical roles in stomatal movement, vegetative growth, and pollen production in *Arabidopsis thaliana*. *J. Exp. Bot.* 62 5179–5189. 10.1093/jxb/err223 21813794 PMC3193020

