# Supplementary material for: Identification and Profiling of microRNAs Expressed in Elongating Cotton Fibers Using Small RNA Deep Sequencing
Source: Front Plant Sci. 2016 Nov 17;7:1722. doi: 10.3389/fpls.2016.01722 (PMC5112280; doi:10.3389/fpls.2016.01722)

Figure S1. Secondary structure of 46 novel miRNA precursors identified in this study. Mature miRNAs are highlighted in red.

GhmiRna01-1

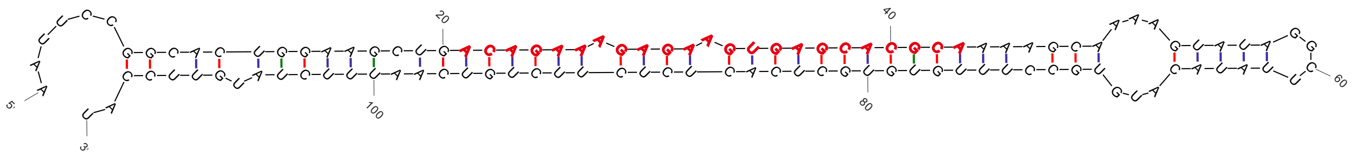

GhmiRna01-2

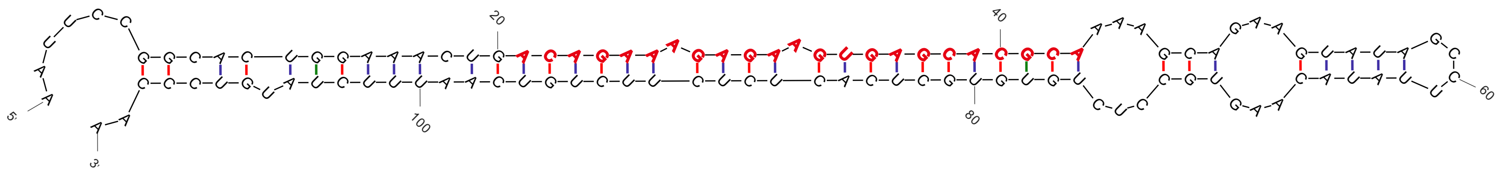

GhmiRna02

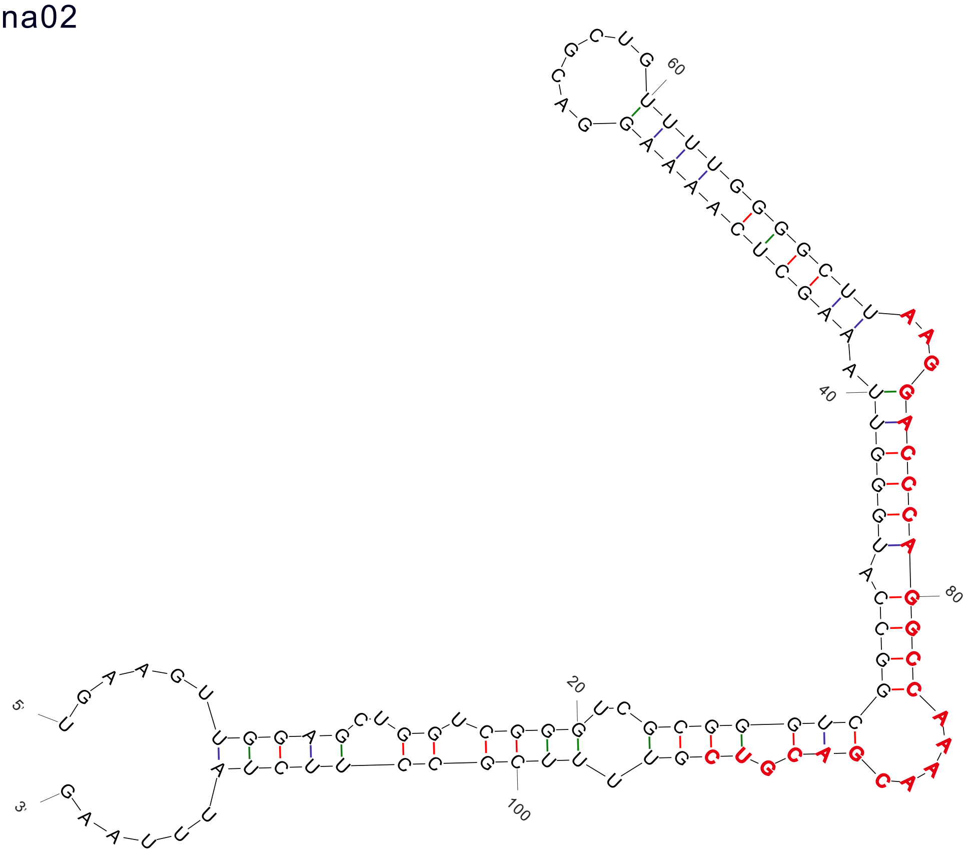

GhmiRna03

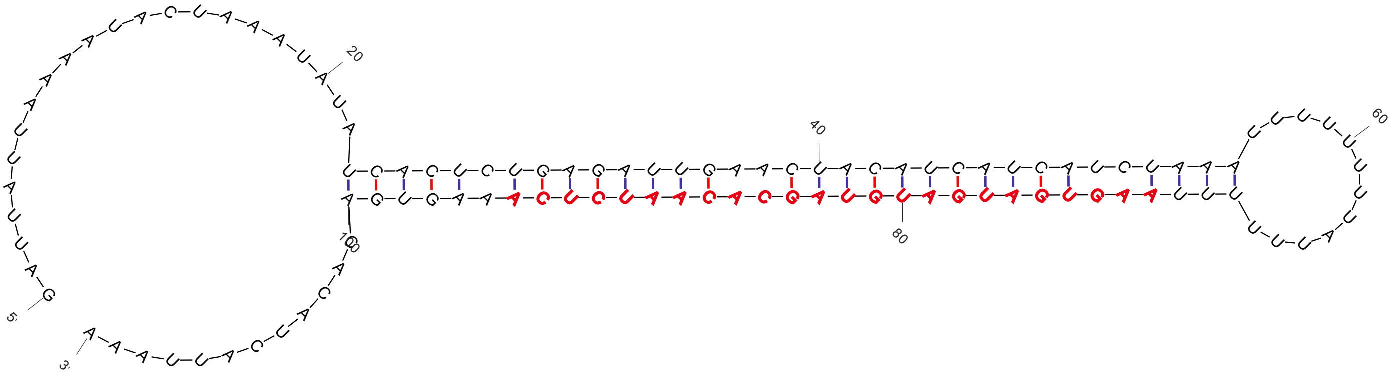

GhmiRna04

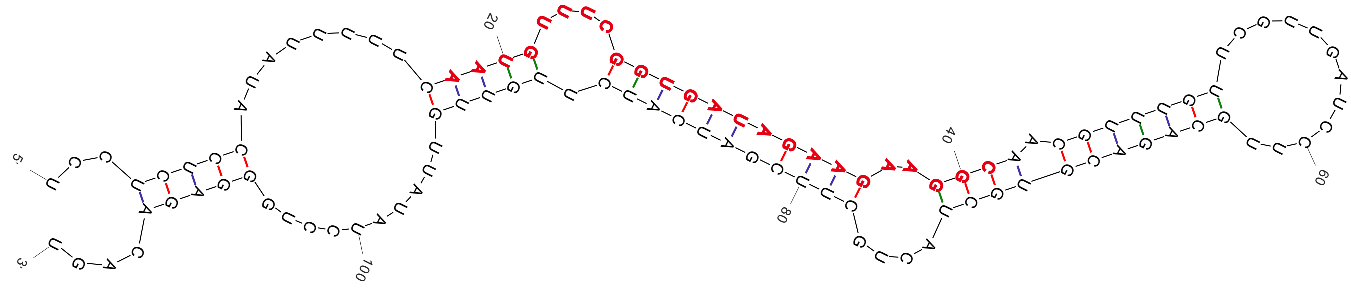

GhmiRna05

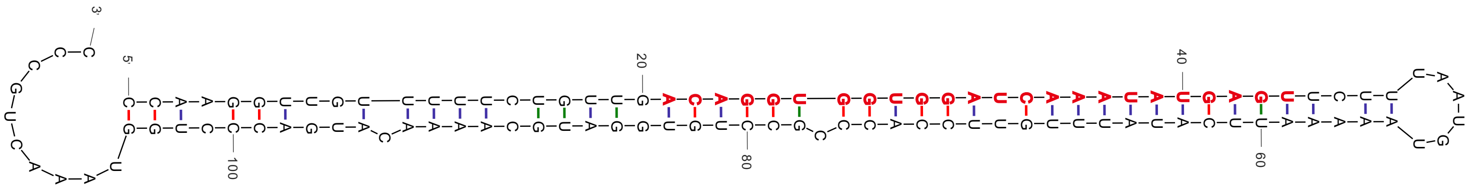

GhmiRna06

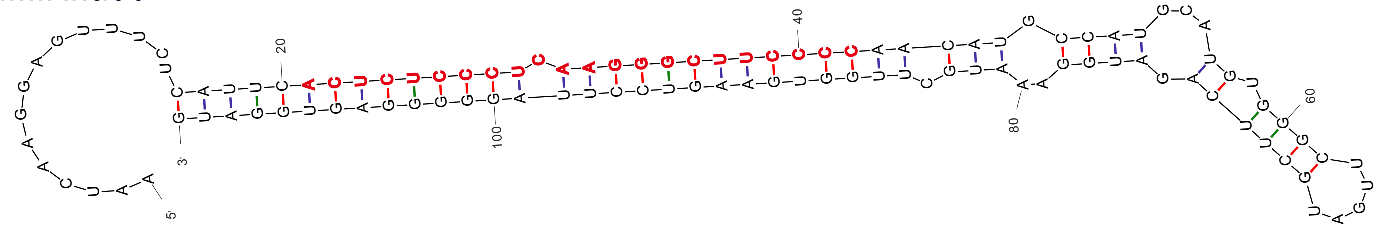

GhmiRna07

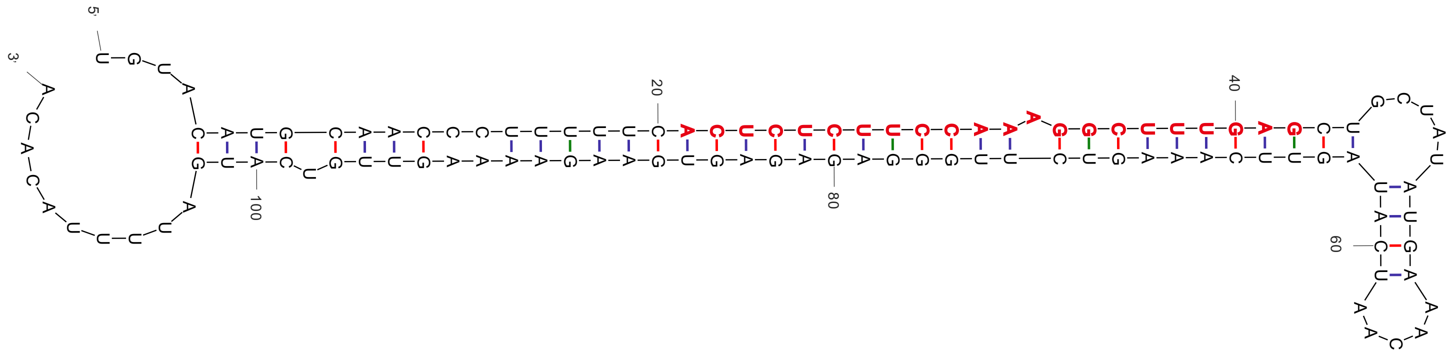

GhmiRna08

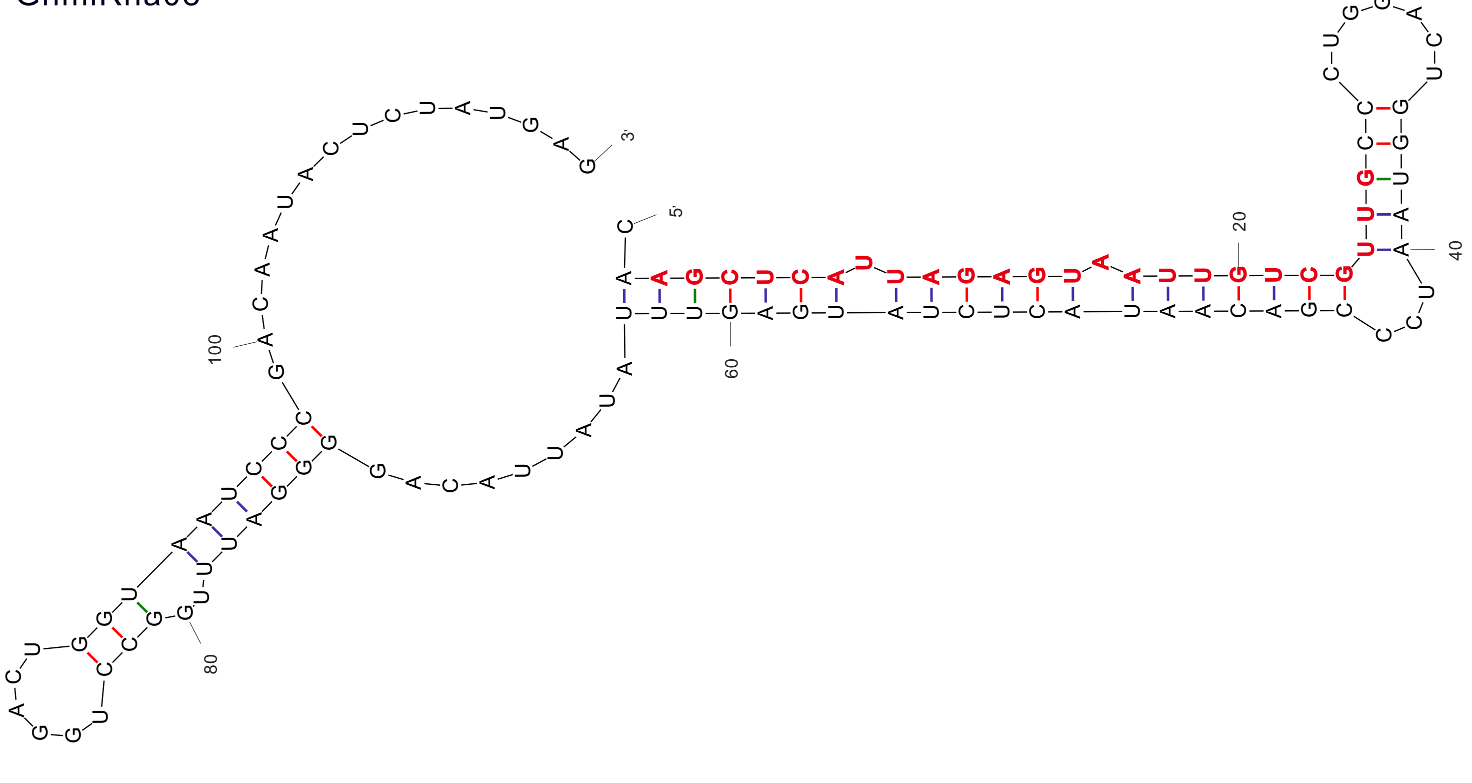

GhmiRna09

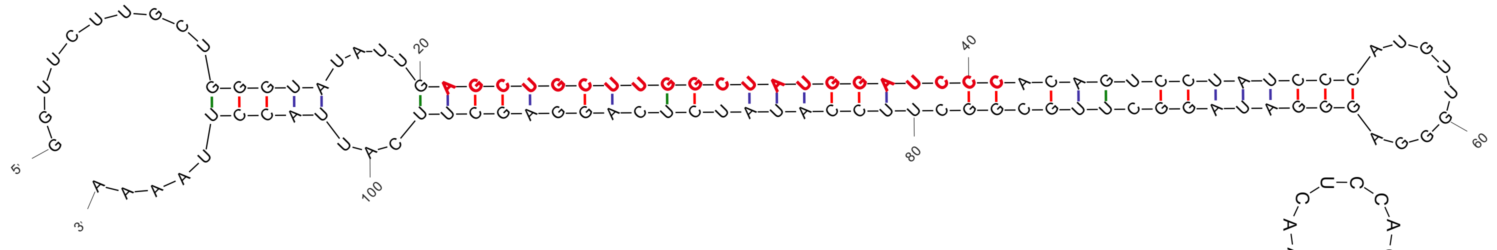

GhmiRna10

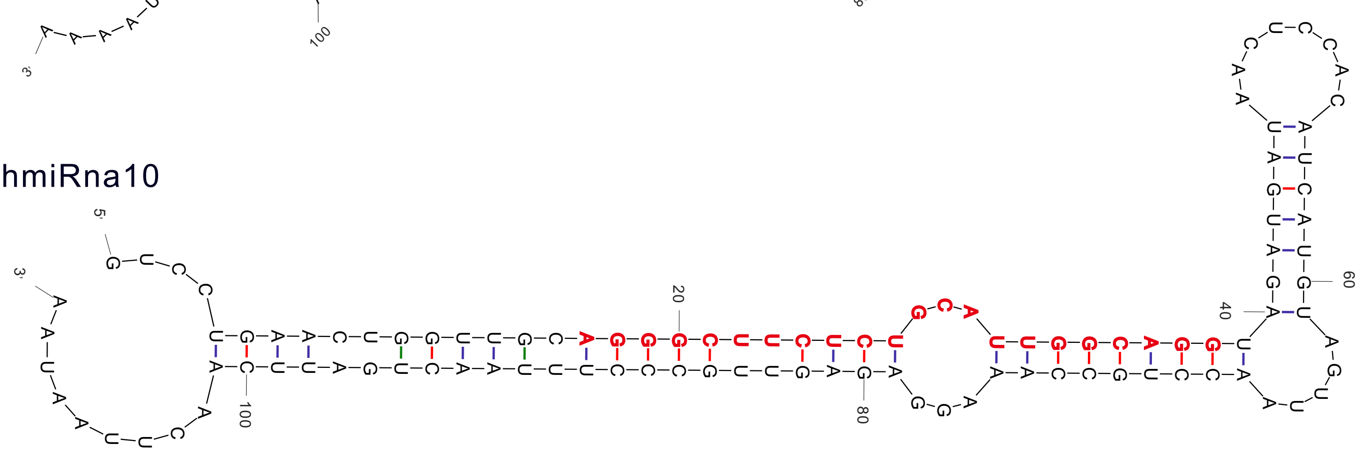

GhmiRna11

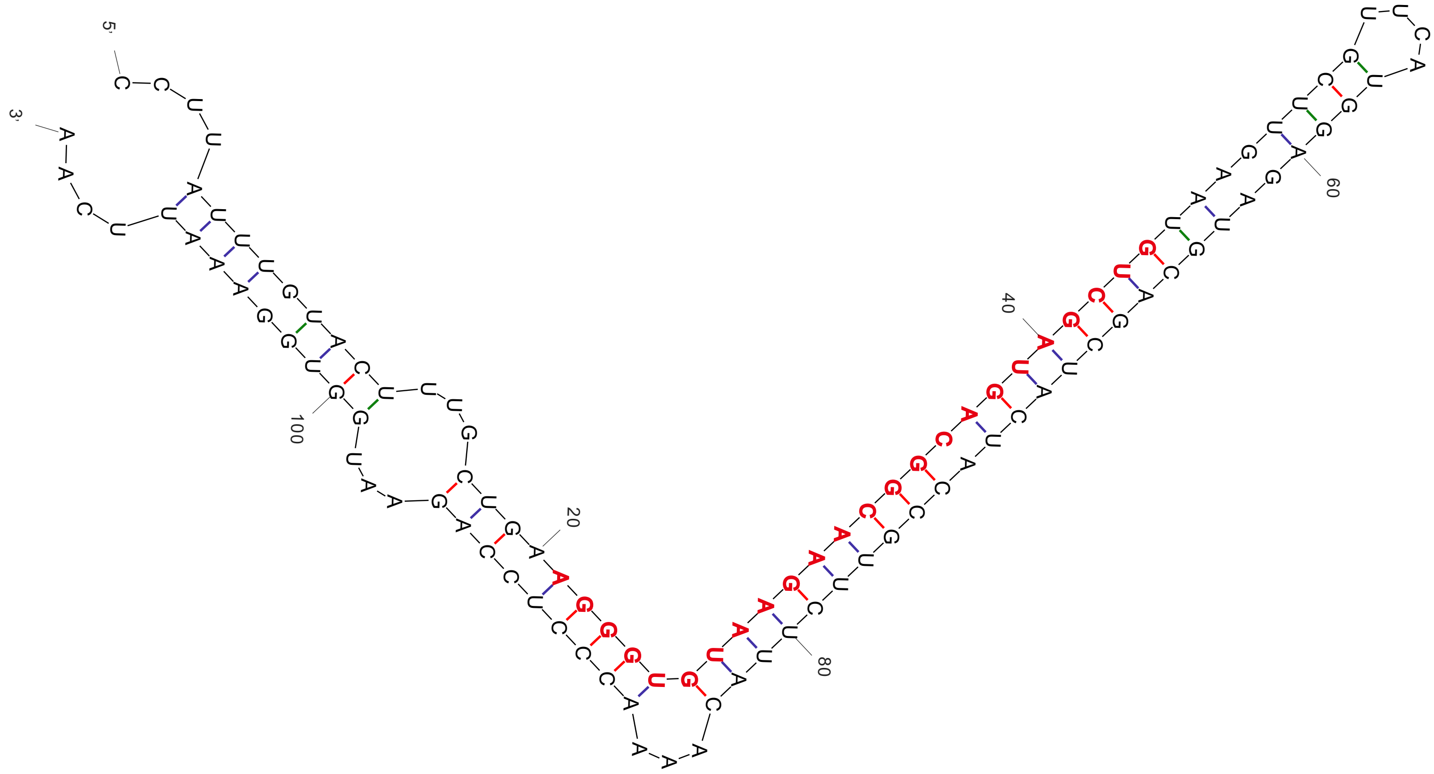

GhmiRna12

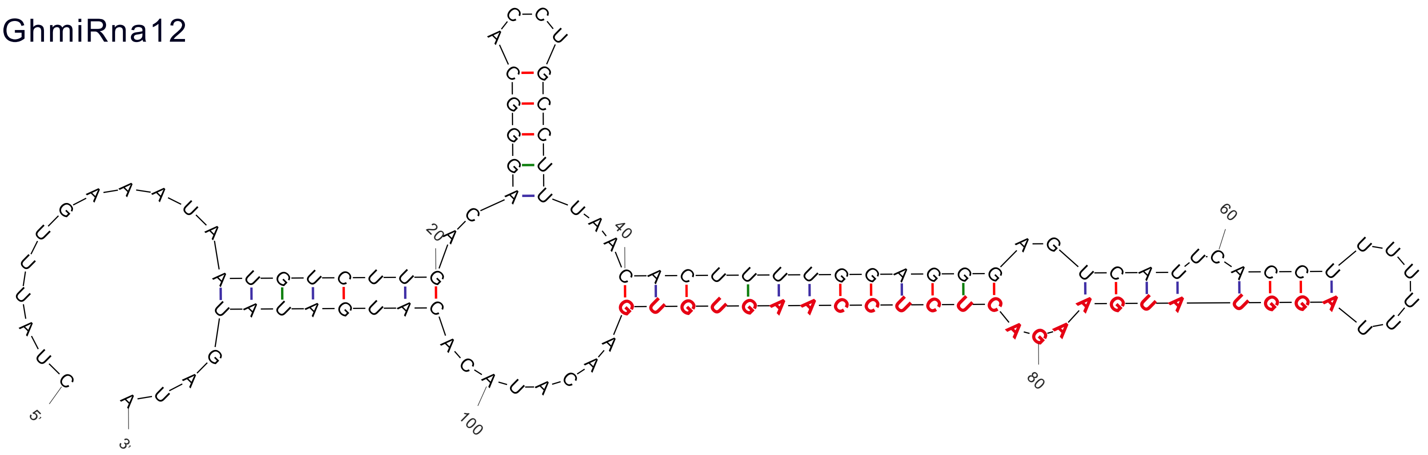

GhmiRna13

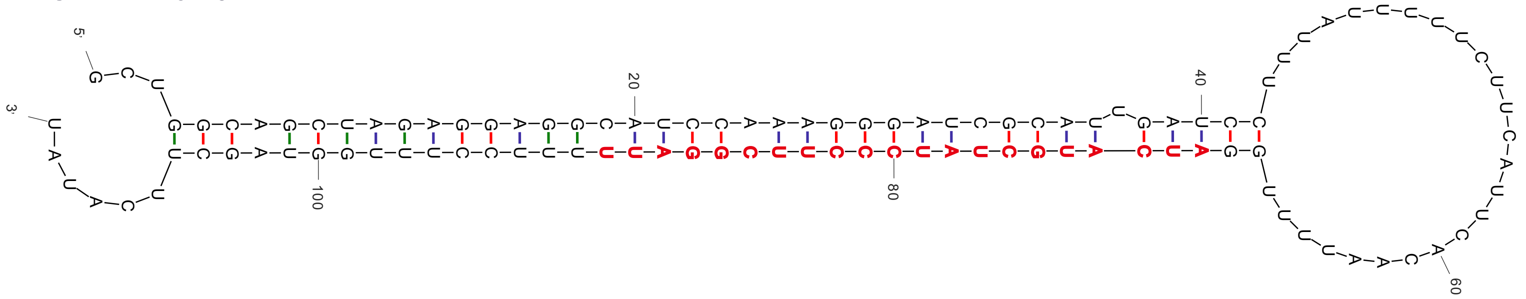

GhmiRna14

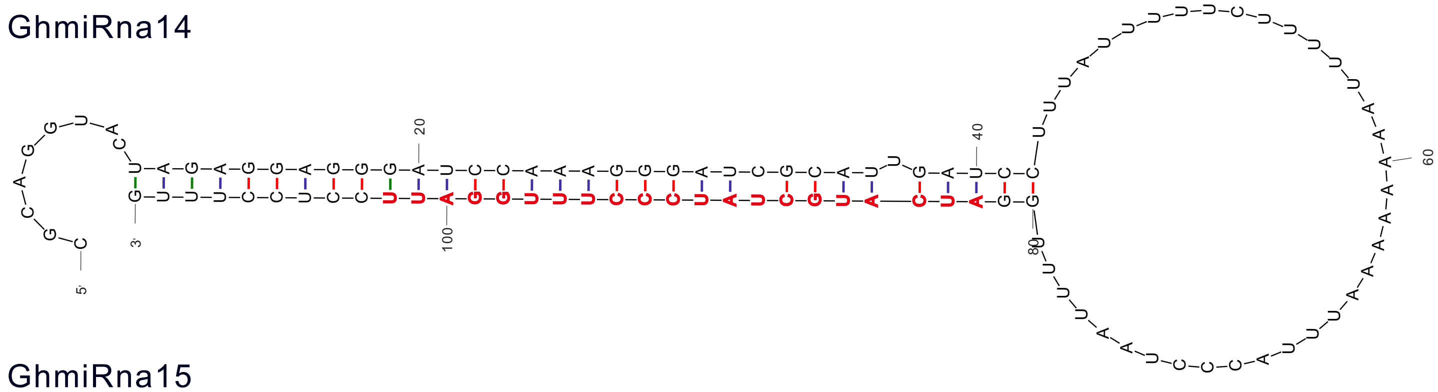

GhmiRna15

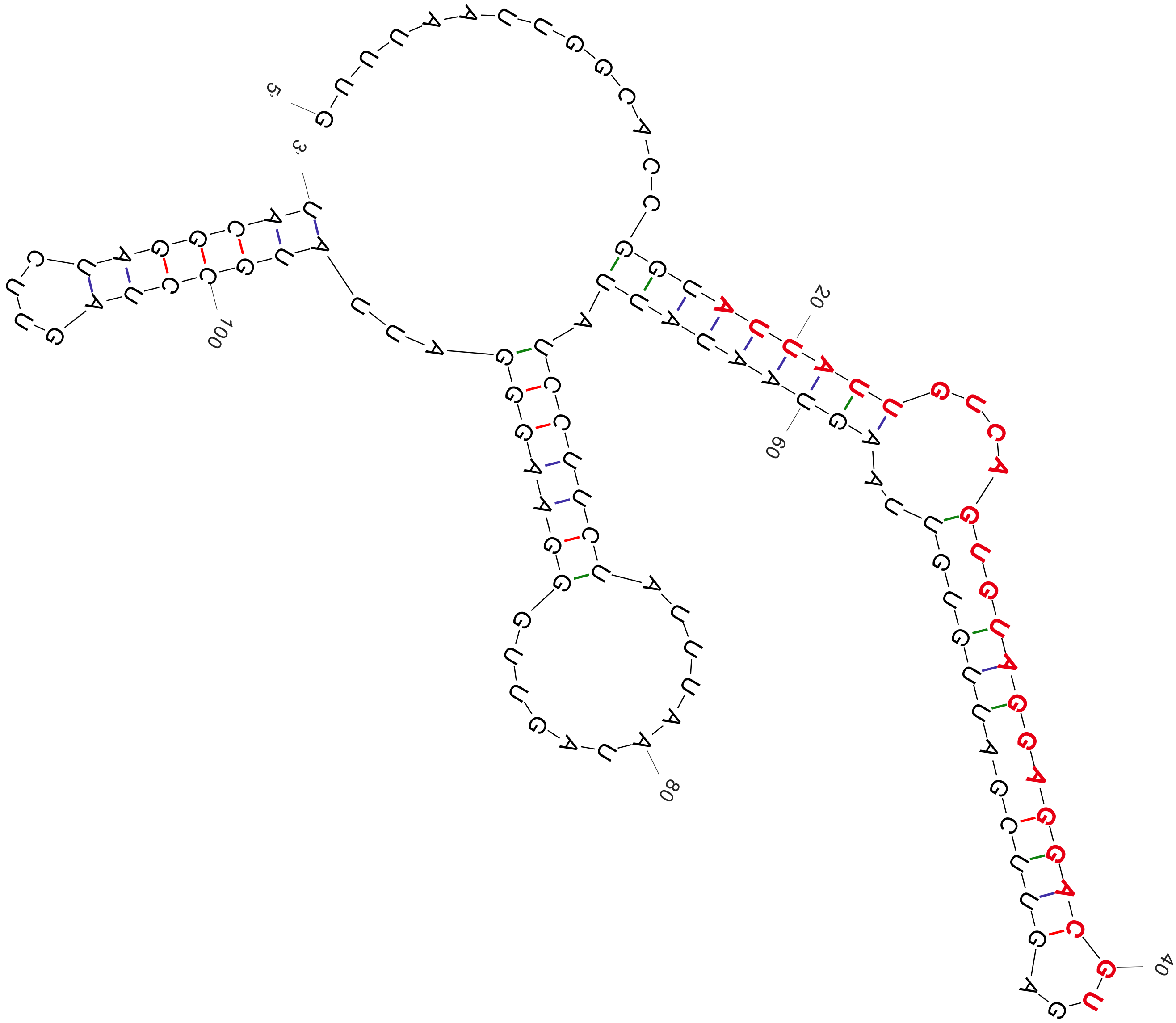

GhmiRna16

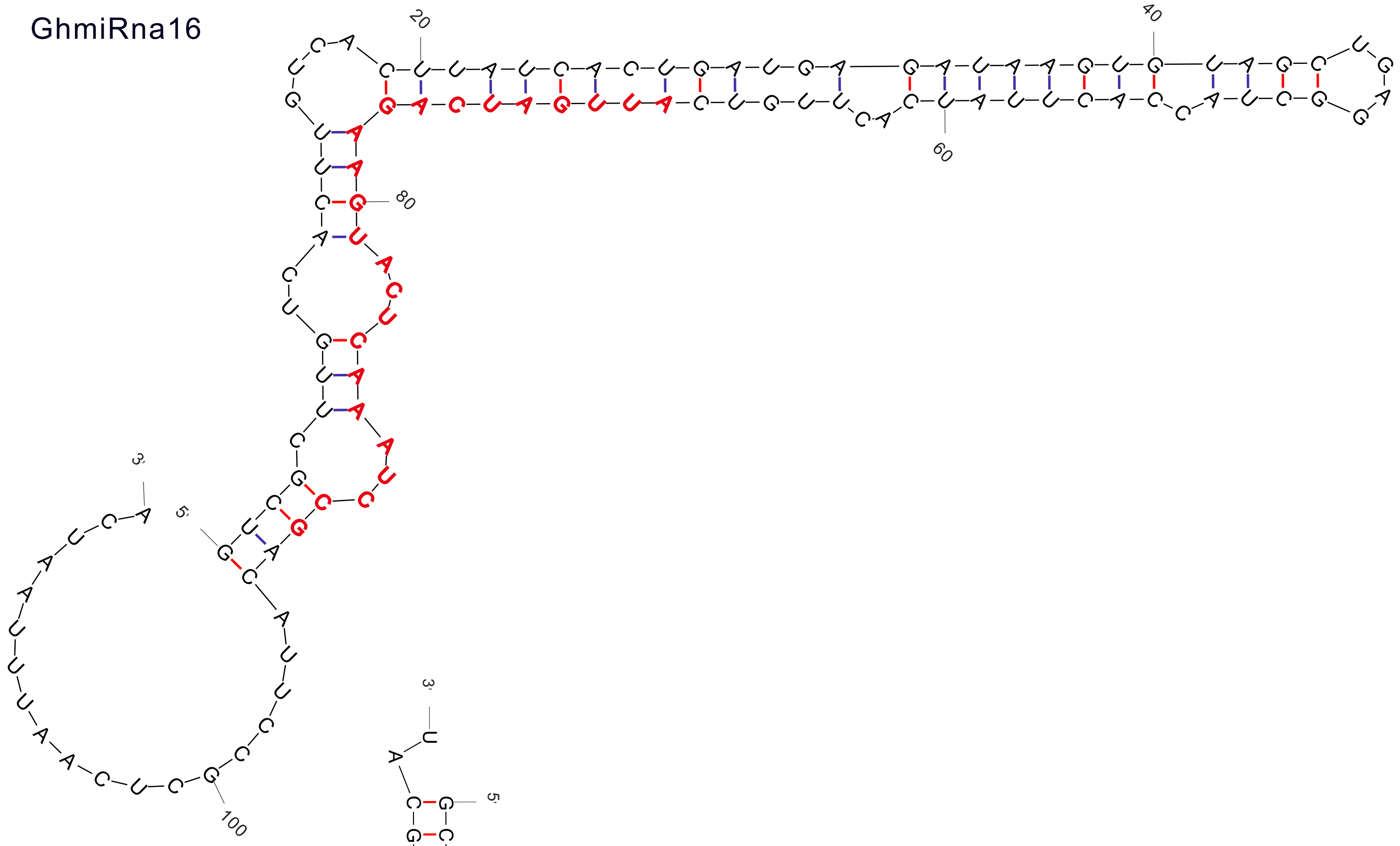

GhmiRna17

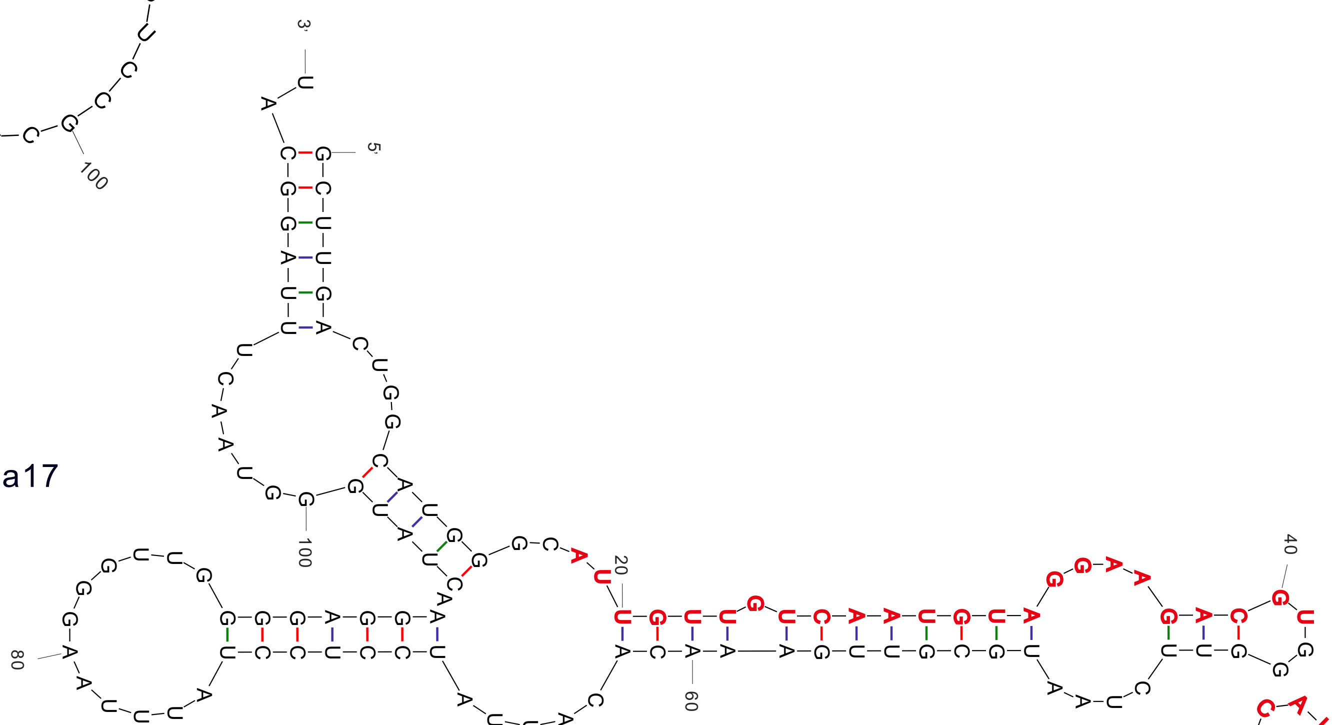

GhmiRna18

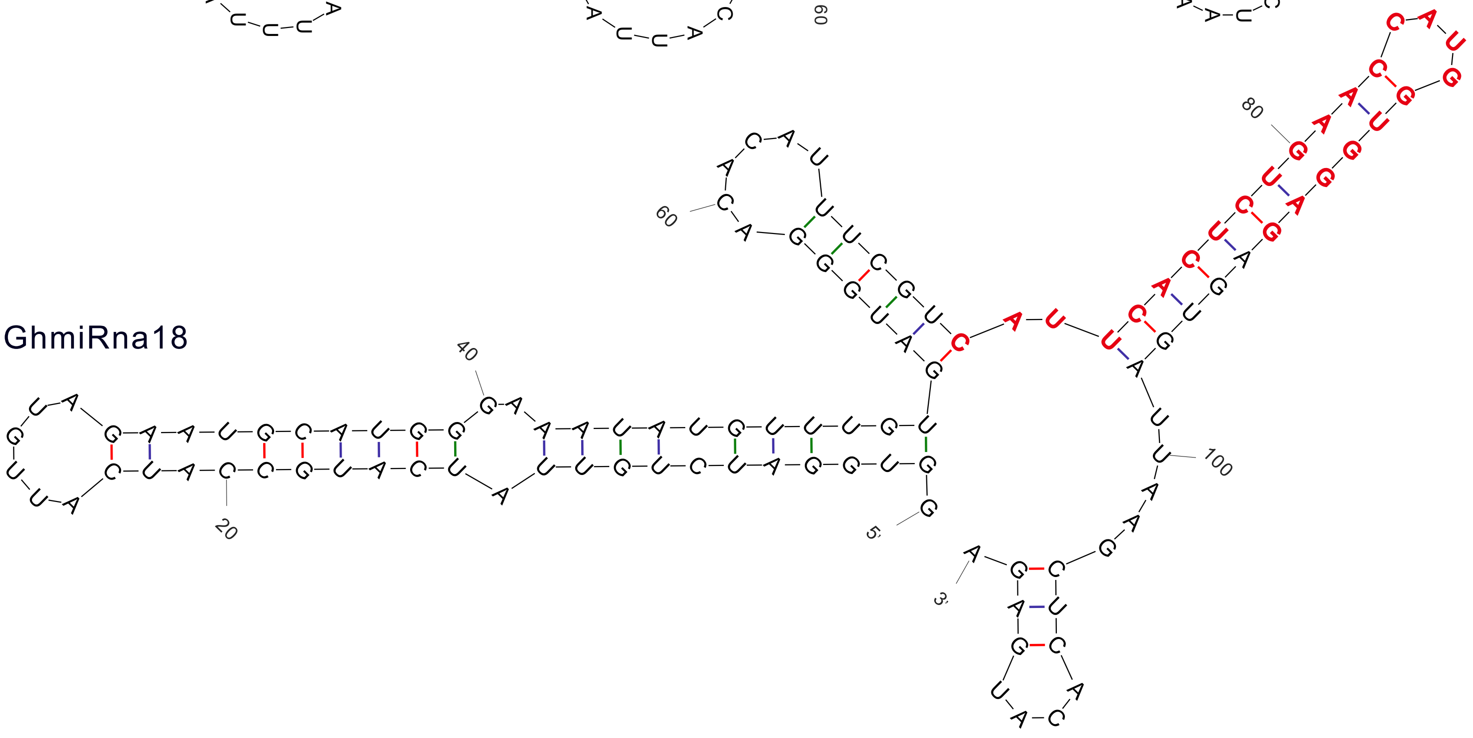

GhmiRna19

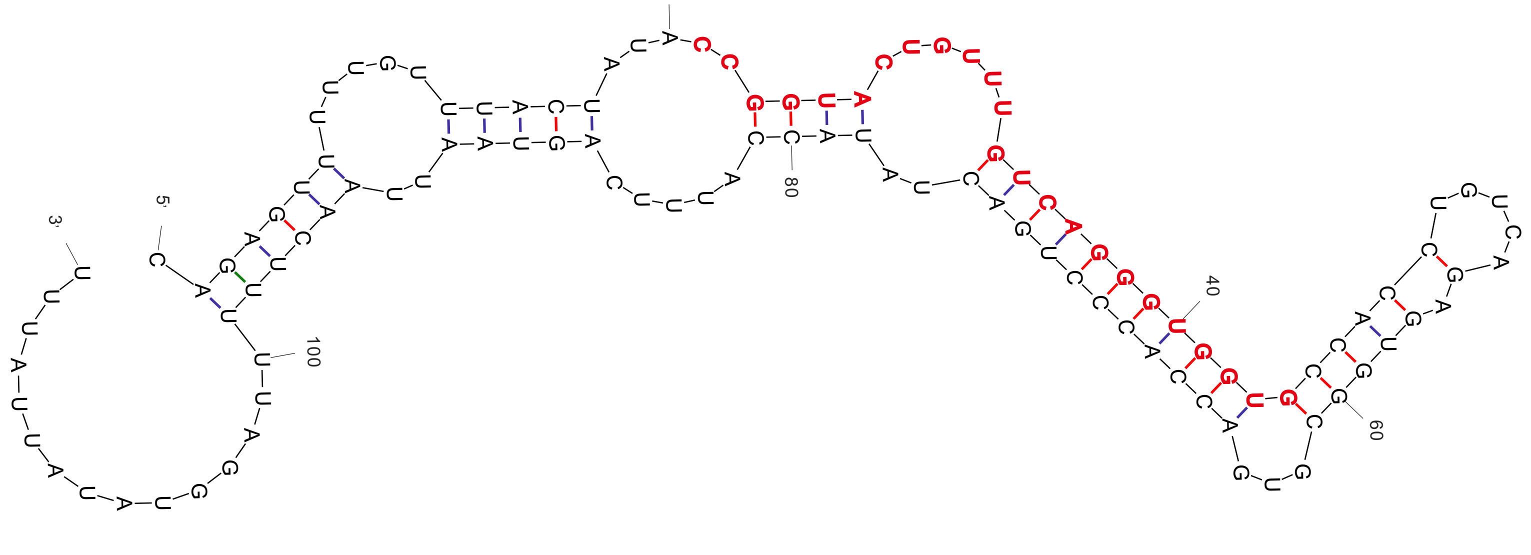

GhmiRna20

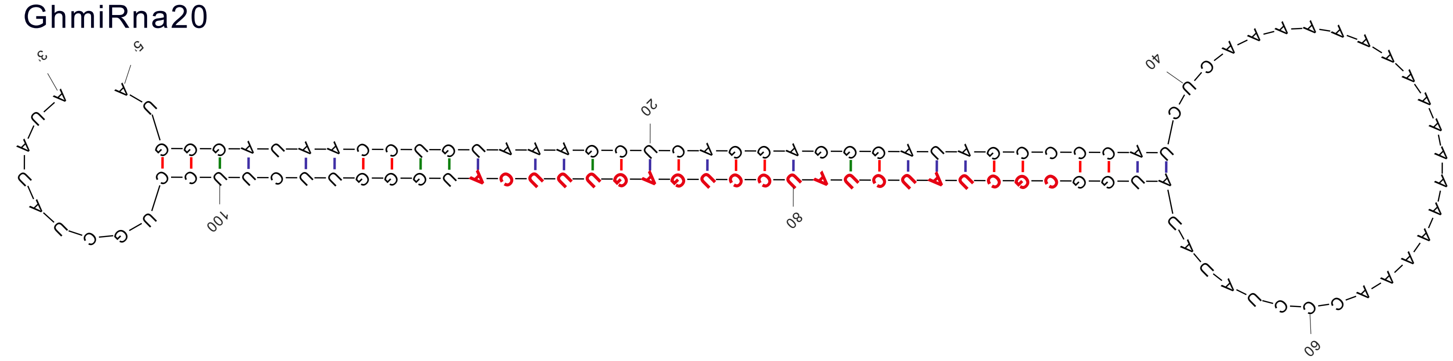

GhmiRna21

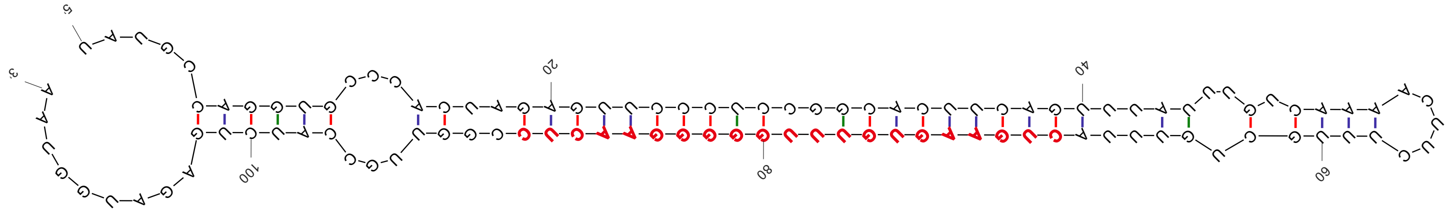

GhmiRna22

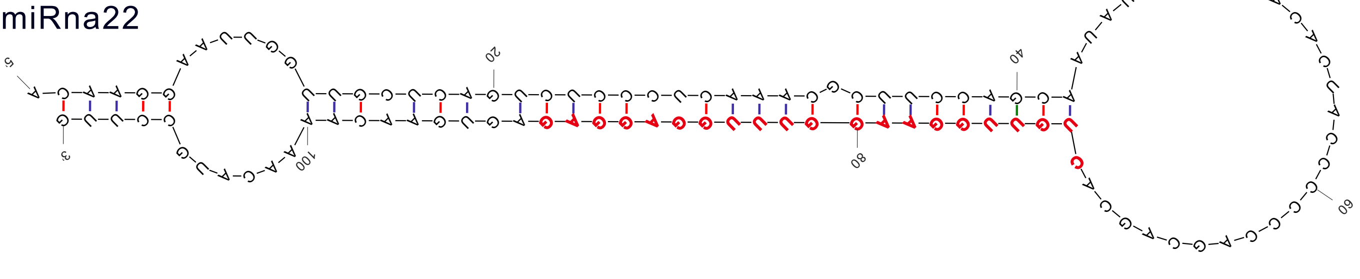

GhmiRna23

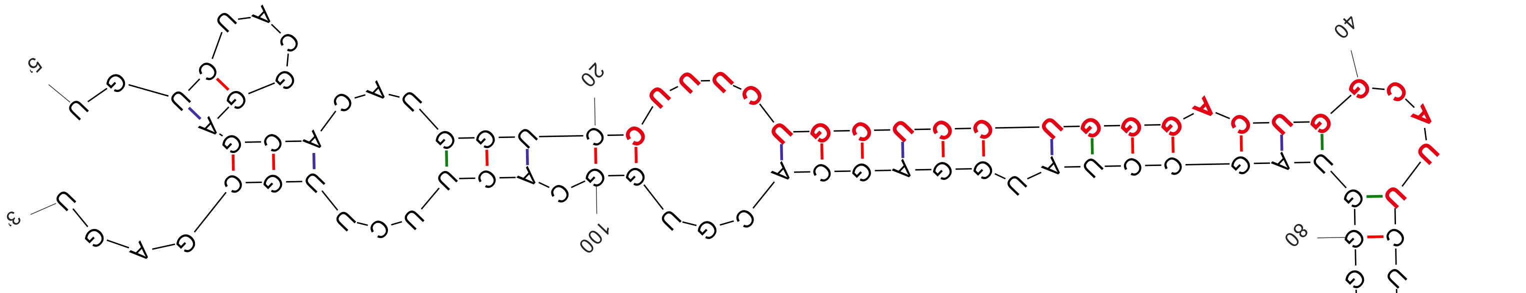

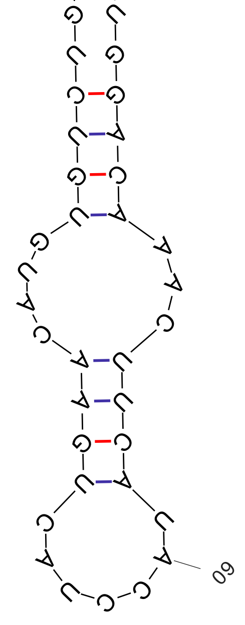

GhmiRna24

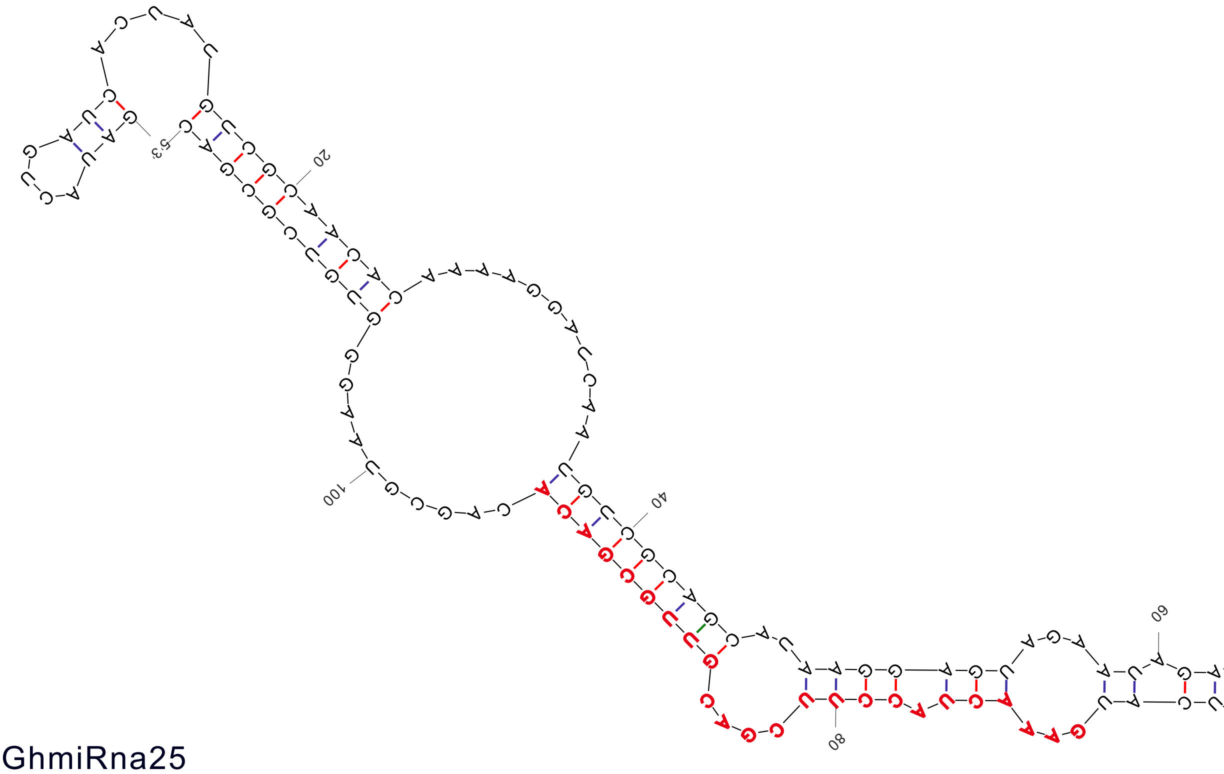

GhmiRna25

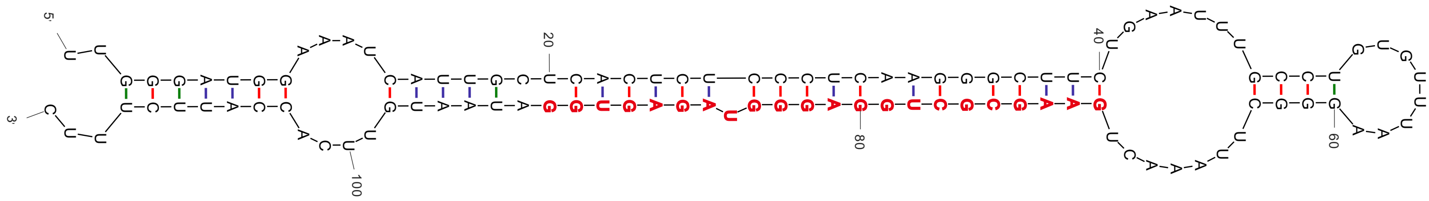

GhmiRna26

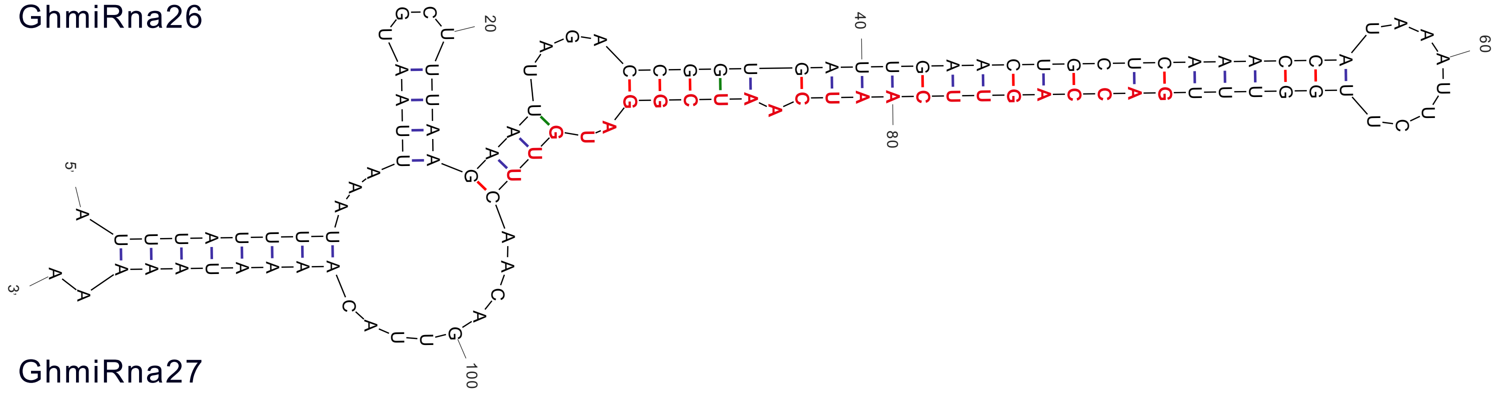

GhmiRna27

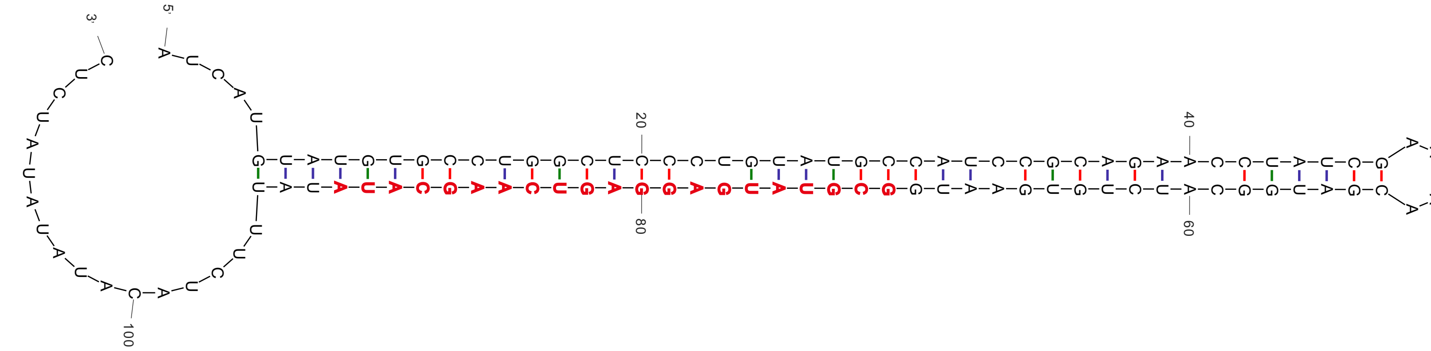

GhmiRna28

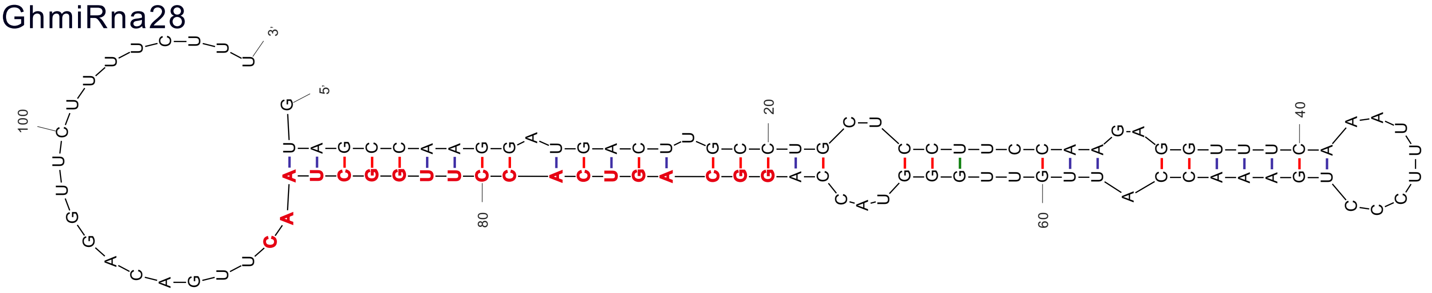

GhmiRna29

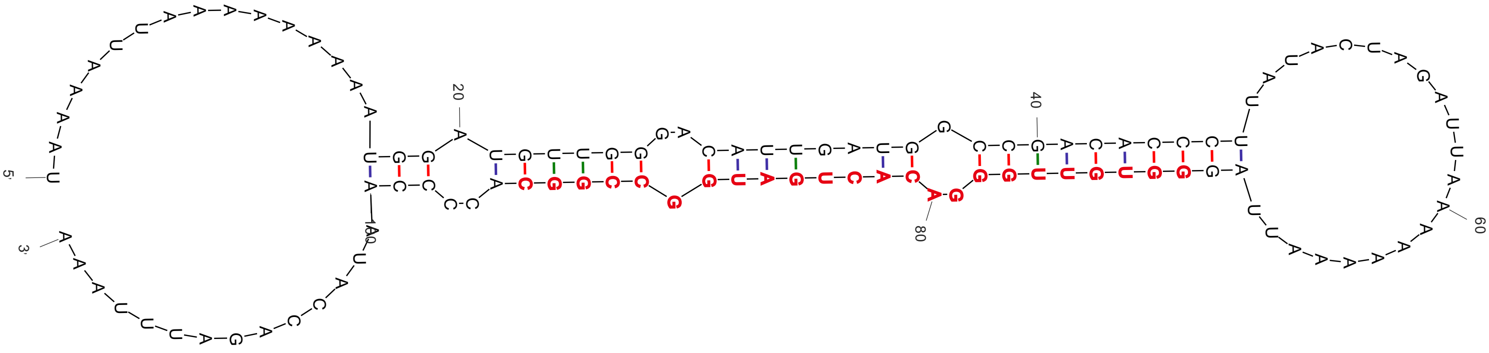

GhmiRna30

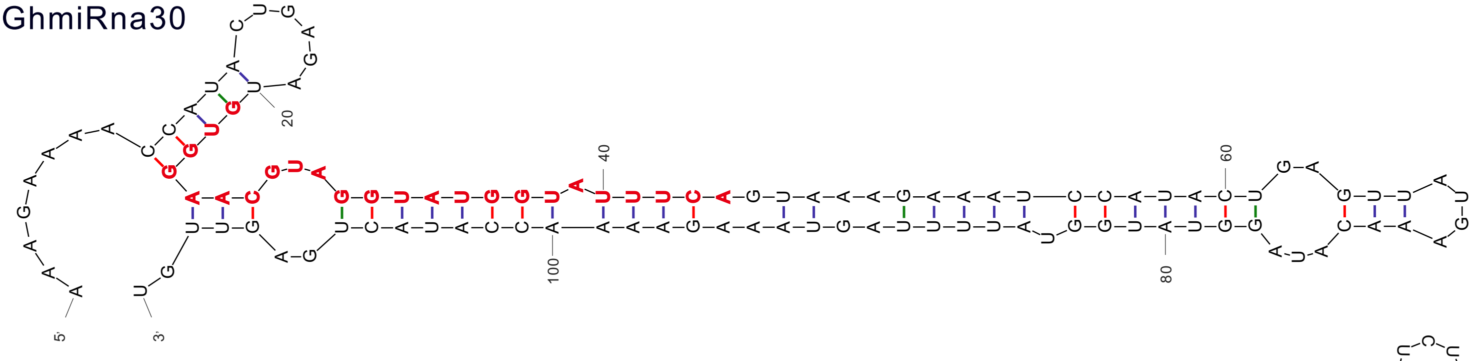

GhmiRna31

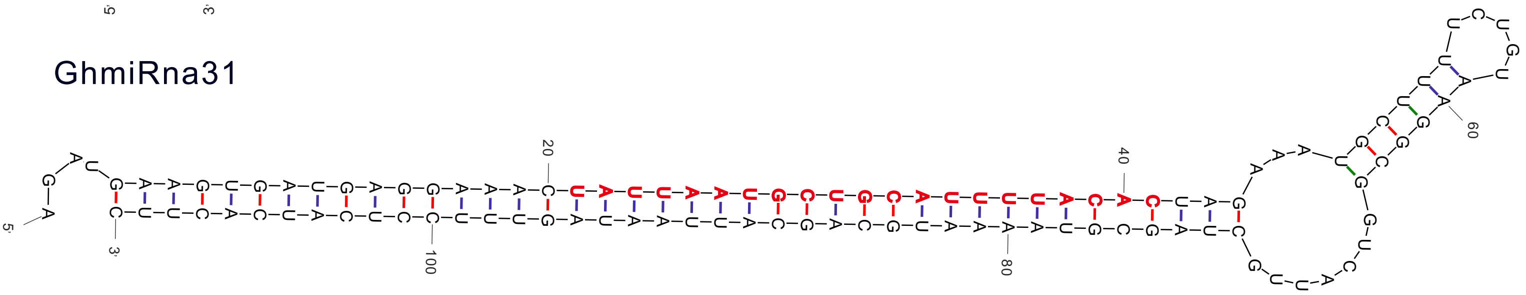

GhmiRna32

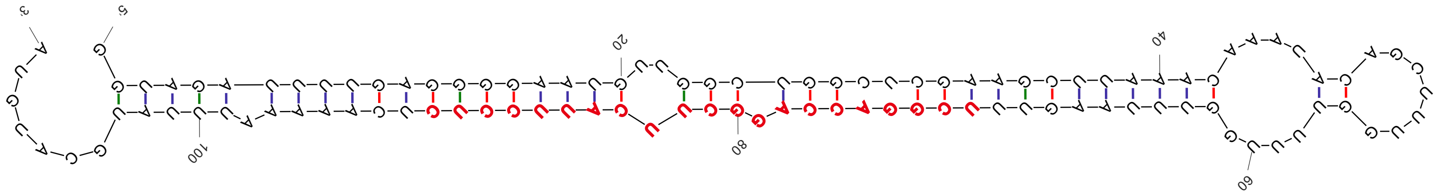

GhmiRna33

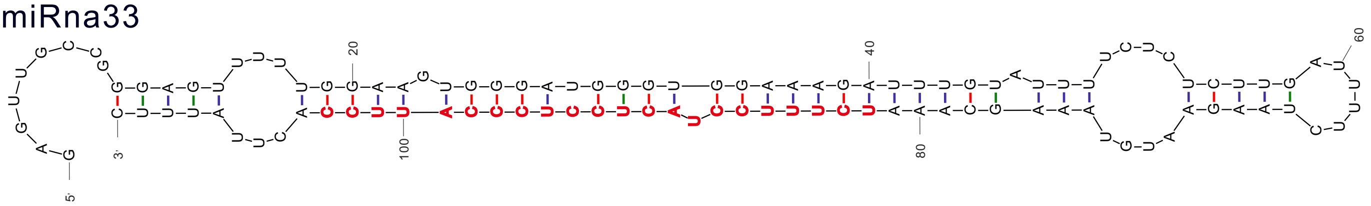

GhmiRna34

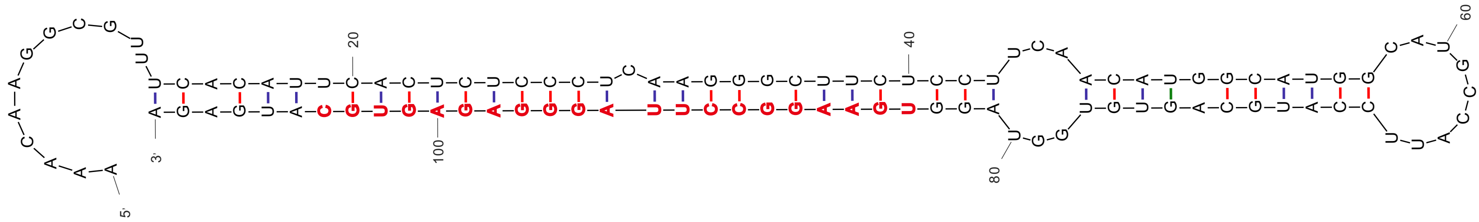

GhmiRna35

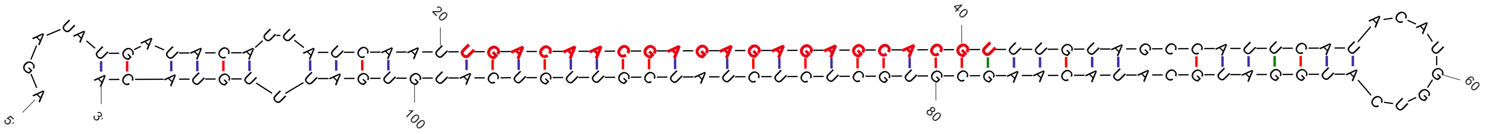

GhmiRna36

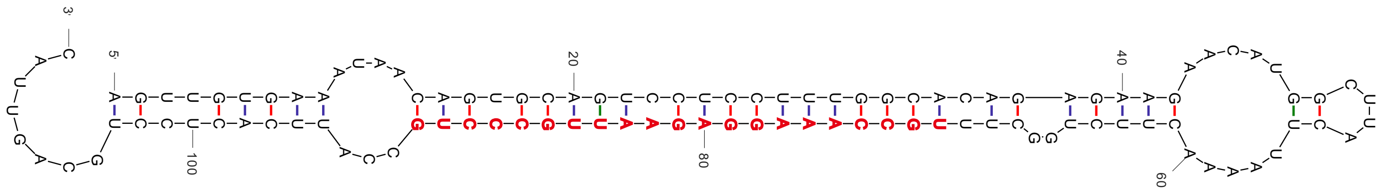

GhmiRna37

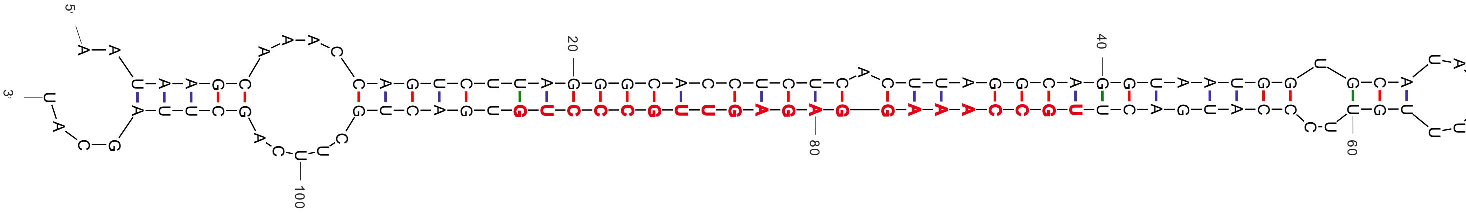

GhmiRna38

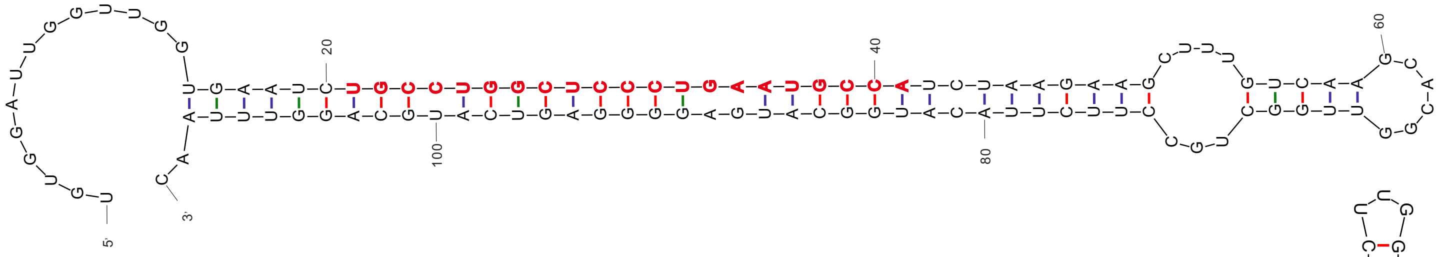

GhmiRna39

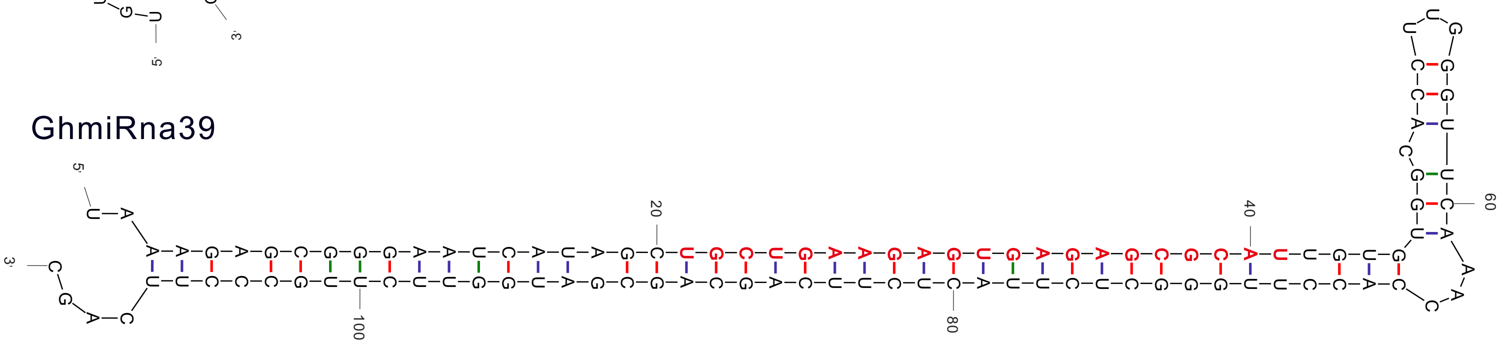

GhmiRna40

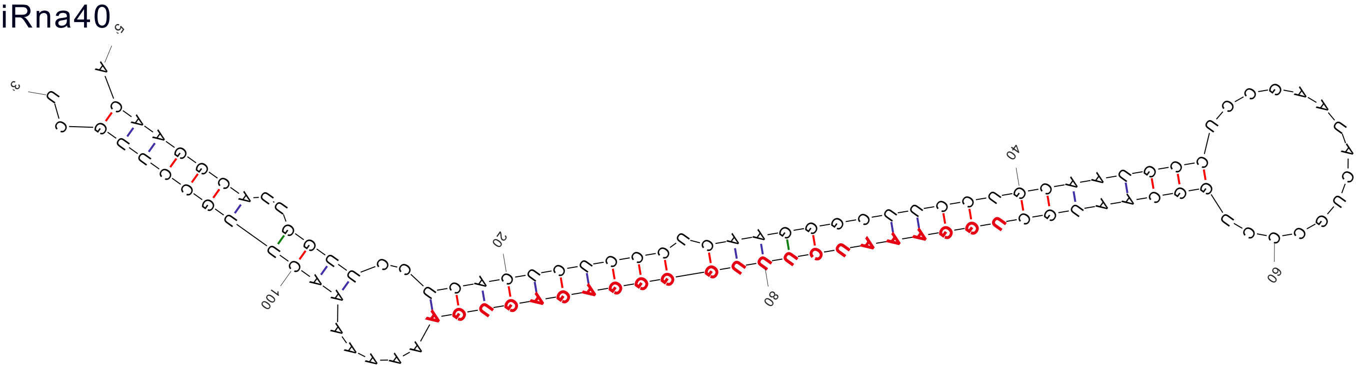

GhmiRna41

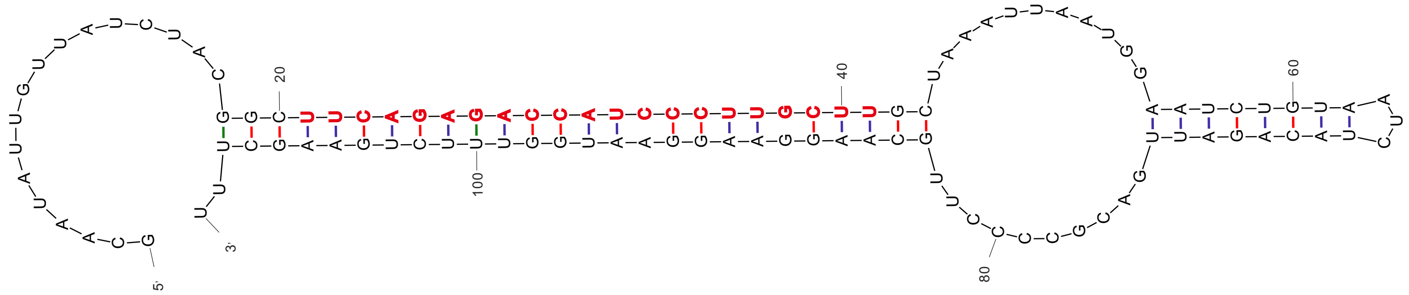

GhmiRna42

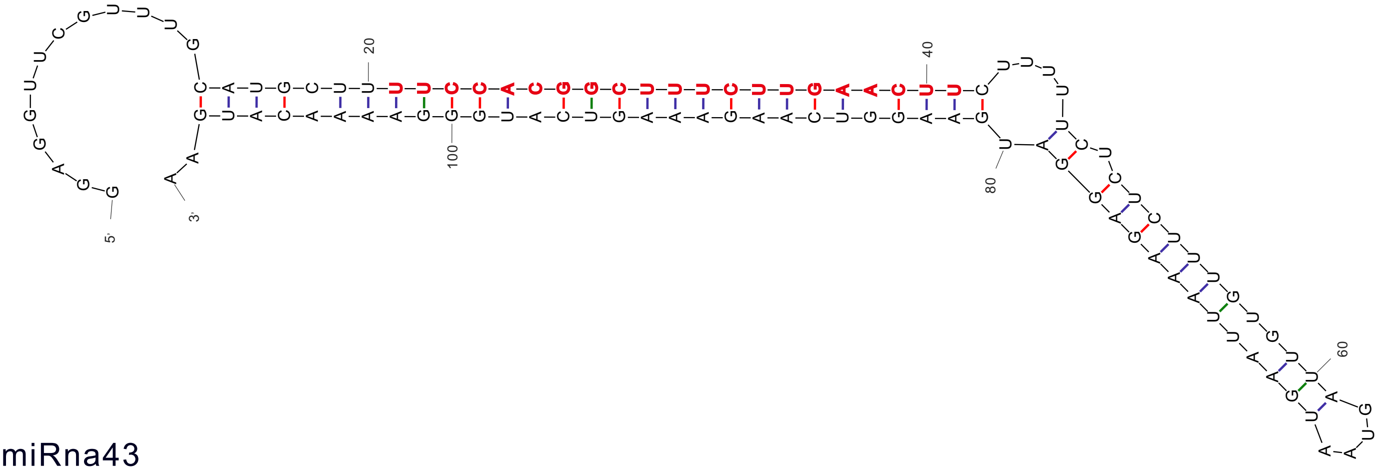

GhmiRna43

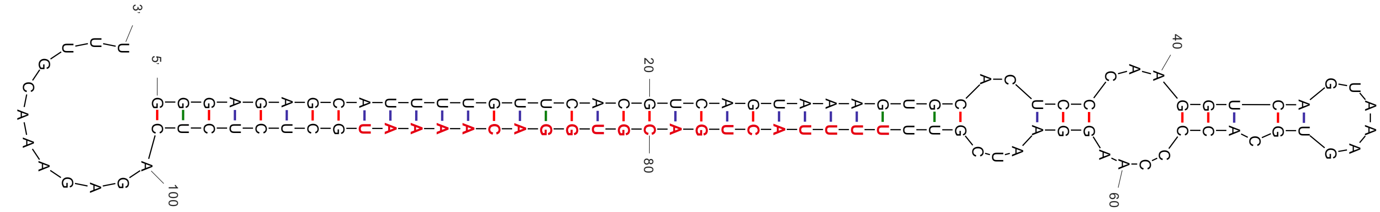

GhmiRna44

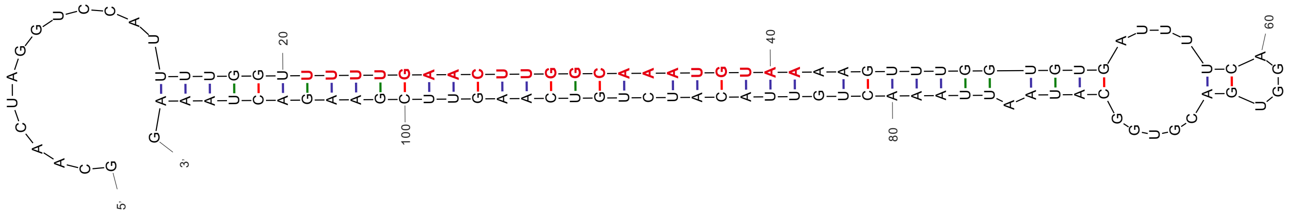

GhmiRna45

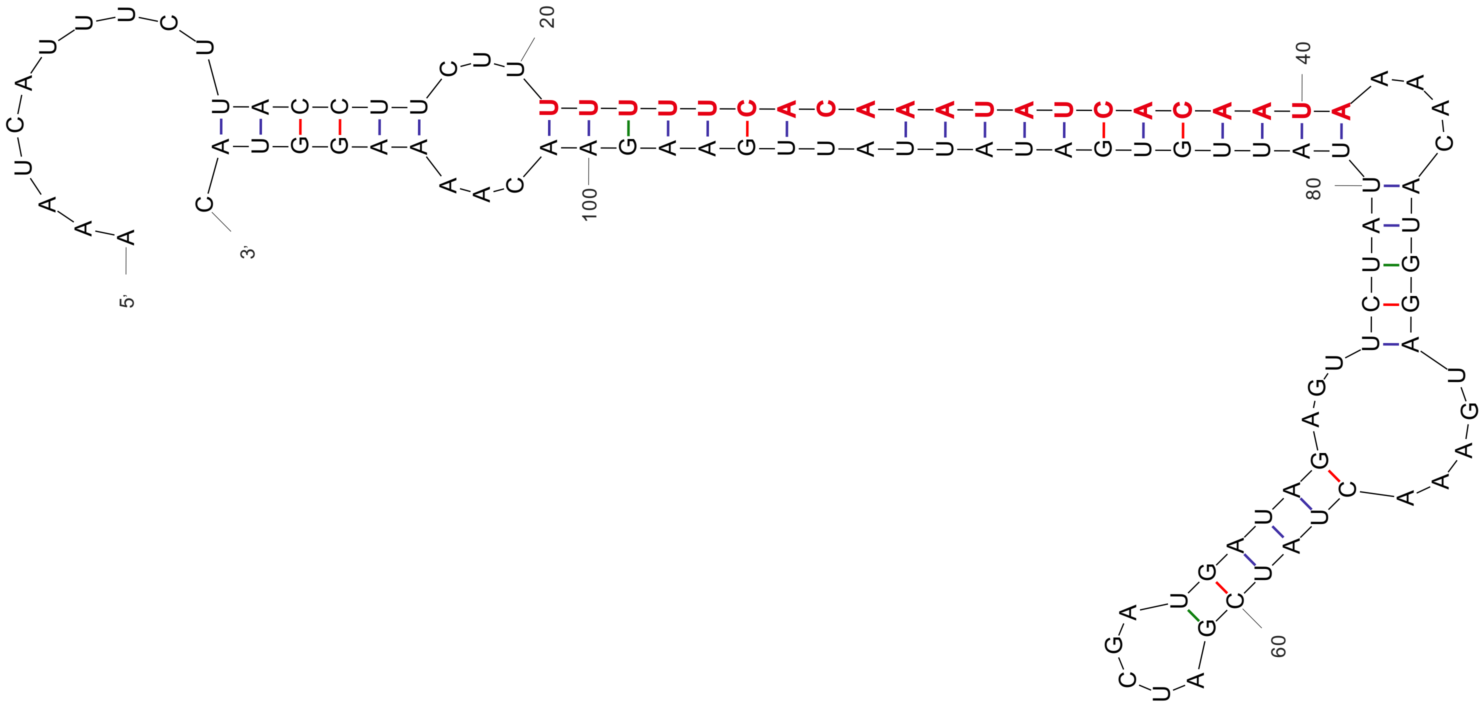

Supplement: Supplementary file 2 [file Image_1.PDF]
