# Supplementary material for: Identification and Profiling of microRNAs Expressed in Elongating Cotton Fibers Using Small RNA Deep Sequencing
Source: Front Plant Sci. 2016 Nov 17;7:1722. doi: 10.3389/fpls.2016.01722 (PMC5112280; doi:10.3389/fpls.2016.01722)

Figure S2. Venn diagram illustrates the numbers of common and uniquely expressed known and novel miRNAs in the cotton fibers (5, 10, 15 and 20 DPA). (A) The distribution of all known miRNAs identified in the study. (B) The distribution of all novel miRNAs identified in the study. (C) The distribution of 48 differentially expressed known miRNAs. (D) The distribution of 16 differentially expressed novel miRNAs.

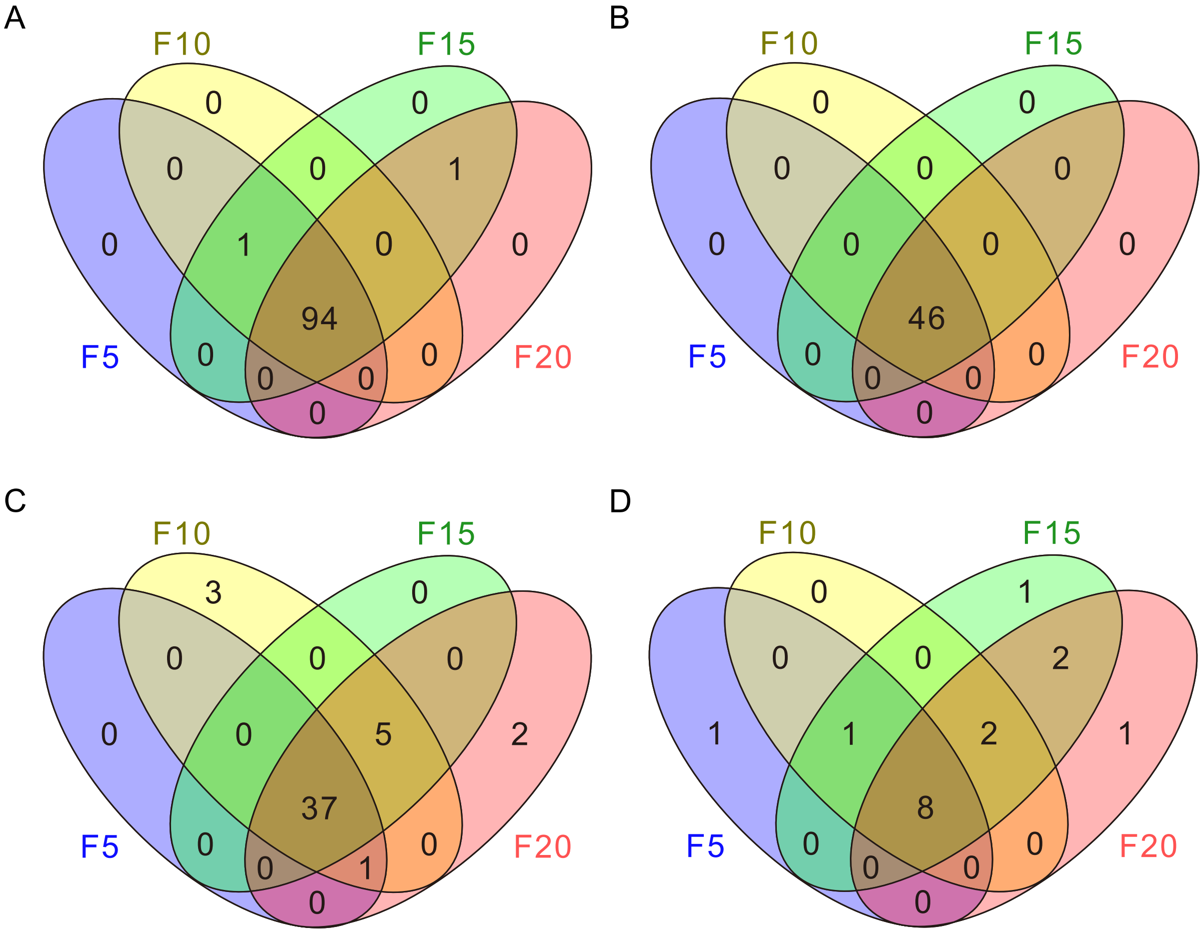

Supplement: Supplementary file 3 [file Image_2.PDF]
