# Supplementary material for: Identification and Profiling of microRNAs Expressed in Elongating Cotton Fibers Using Small RNA Deep Sequencing
Source: Front Plant Sci. 2016 Nov 17;7:1722. doi: 10.3389/fpls.2016.01722 (PMC5112280; doi:10.3389/fpls.2016.01722)

Figure S3. RNA-seq differentially expressed analysis of nine miRNA target genes during cotton fiber elongation. Color scale represents log_2-based fold changes of fragments per kilobase of exon per million fragments mapped (FPKM).

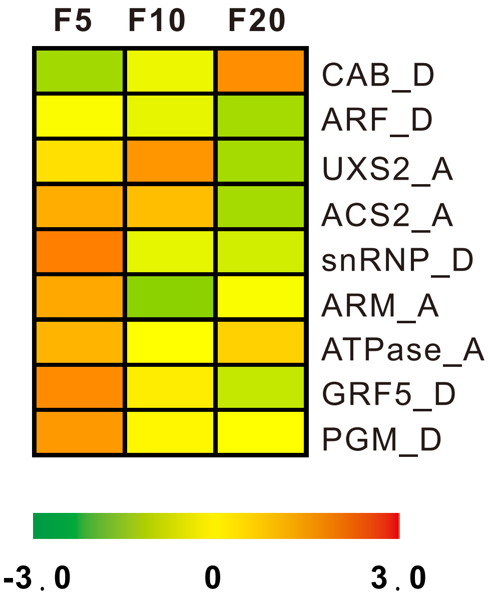

Supplement: Supplementary file 4 [file Image_3.PDF]
